# A highly conserved A-to-I RNA editing event within the glutamate-gated chloride channel GluClα is necessary for olfactory-based behaviors in *Drosophila*

**DOI:** 10.1126/sciadv.adi9101

**Published:** 2024-09-04

**Authors:** Hila Zak, Eyal Rozenfeld, Mali Levi, Patricia Deng, David Gorelick, Hadar Pozeilov, Shai Israel, Yoav Paas, Yoav Paas, Jin Billy Li, Moshe Parnas, Galit Shohat-Ophir

**Affiliations:** ^1^ The Mina & Everard Goodman Faculty of Life Sciences, Bar-Ilan University, Ramat Gan 5290002, Israel.; ^2^ Bar-Ilan University, Ramat Gan 5290002, Israel.; ^3^ The Nanotechnology Institute, Bar-Ilan University, Ramat Gan 5290002, Israel.; ^4^ Department of Physiology and Pharmacology, Sackler School of Medicine, Tel Aviv University, Tel Aviv 69978, Israel.; ^5^ Sagol School of Neuroscience, Tel Aviv University, Tel Aviv 69978, Israel.; ^6^ Department of Genetics, Stanford University, Stanford, CA 94305, USA.

## Abstract

A-to-I RNA editing is a cellular mechanism that generates transcriptomic and proteomic diversity, which is essential for neuronal and immune functions. It involves the conversion of specific adenosines in RNA molecules to inosines, which are recognized as guanosines by cellular machinery. Despite the vast number of editing sites observed across the animal kingdom, pinpointing critical sites and understanding their in vivo functions remains challenging. Here, we study the function of an evolutionary conserved editing site in *Drosophila*, located in glutamate-gated chloride channel (*GluCl*α). Our findings reveal that flies lacking editing at this site exhibit reduced olfactory responses to odors and impaired pheromone-dependent social interactions. Moreover, we demonstrate that editing of this site is crucial for the proper processing of olfactory information in projection neurons. Our results highlight the value of using evolutionary conservation as a criterion for identifying editing events with potential functional significance and paves the way for elucidating the intricate link between RNA modification, neuronal physiology, and behavior.

## Teaser

A conserved A-to-I RNA editing site in glutamate-gated chloride channel GluClα is necessary for olfactory behaviors in *Drosophila.*

## INTRODUCTION

Adenosine-to-inosine (A-to-I) RNA editing, catalyzed by ADAR enzymes, is a ubiquitous mechanism that generates transcriptomic and proteomic diversity in metazoans (*1*, *2*). Most of the RNA editing events in mammals occur in noncoding parts of the transcriptome by ADAR1 and serve to prevent aberrant immune responses toward self–double-stranded RNAs (dsRNAs) (*3*–*6*). Only a small fraction of editing events occur in protein-coding sequences mainly by ADAR2, where the deamination of adenosine to inosines introduces nonsynonymous substitutions (known as recoding events) that produce different protein isoforms from a single DNA sequence (*7*–*12*). The binding of ADAR to dsRNA structures containing target sequence and an editing complementary sequence (ECS) promotes the conversion of specific adenosines within the target sequence into inosines, which are subsequently recognized by the translation machinery as guanosine (G) (*11*). Most recoding events occur within genes that function in neurons, accounting for neuronal deficits exhibited by ADAR2 knockout mice (*13*). A central example is an essential recoding event of glutamine to arginine within the M2 domain of AMPA receptor that is necessary for the proper function of the channel, the absence of which causes lethality (*13*–*15*). Another functionally important recoding event is found within the calmodulin-binding IQ domain of the voltage-activated calcium channel (Ca_v_1.3) and functions to regulate the calcium-dependent inactivation kinetics of the channel (*16*), which, in turn, shapes hippocampal plasticity and memory in mice (*16*, *17*).

Recent advances in RNA sequencing and computational analysis facilitated the identification of millions of editing sites in humans (*1*, *3*, *18*–*20*), tens of thousands in mice (*1*), and thousands in *Drosophila* (*9*, *21*–*23*). While the function of RNA editing in *Drosophila melanogaster* resembles that of mammals (*8*, *24*–*32*), most editing events in *D. melanogaster* are predicted to cause nonsynonymous protein-coding changes, making it hard to pinpoint which of their in vivo function to further study. As a consequence, research in the field was limited to studying the function of few editing events at the biochemical level (*12*, *33*–*36*), leaving their physiological function at the whole organism level practically unknown. To bridge this gap, we chose to study a recoding site that shows high evolutionary conservation across several *Drosophila* species and is found within the extracellular domain of glutamate-gated chloride channel (GluClα) (*37*). The high evolutionary conservation of the particular recoding of Isoleucine at position 27 to valine is suggestive of its functional importance (*37*).

**Fig. 1.**
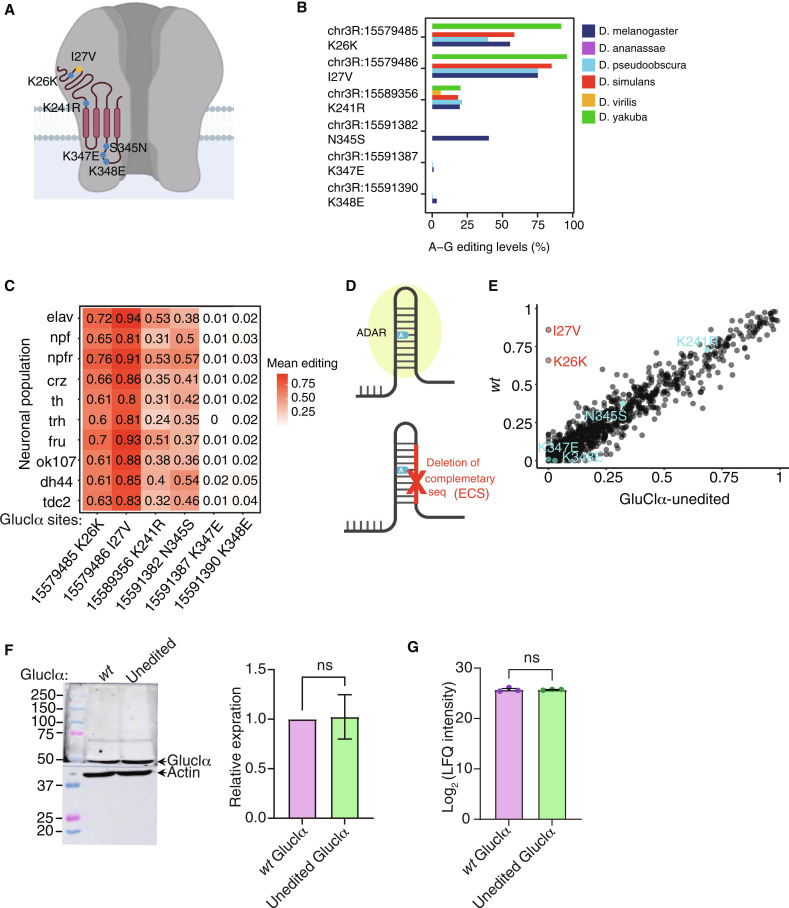
The recoding of Ile^27^ to Val is conserved across *Drosophila* species. (**A**) Schematic representation of the *Drosophila* GluClα channel and the relative location of its A-to-I editing sites. (**B**) Relative editing levels of GluClα-editing sites in RNA extracted from various *Drosophila* species. *n* = 2 per strain. (**C**) Average editing levels of GluClα sites across various neuronal populations in *Drosophila melanogaster* brain (*n* = 3 for each neuronal population). (**D**) Schematic illustration of the approach used to generate GluClα-unedited flies. ADAR binds to a dsRNA structure formed by base pairing between an exon sequence containing the editing site and a complementary intronic sequence (ECS). Deletion of the ECS required for the editing of Ile^27^ was achieved via CRISPR-Cas9. (**E**) Pearson correlation comparing editing patterns of 1766 sites between GluClα-unedited and *wt D. melanogaster* flies; sites within GluClα transcripts are colored (*n =* 3 samples per neuronal population). (**F**) Relative GluClα protein levels extracted from GluClα-unedited and *wt* heads. Left, representative blot (α-GluClα antibodies and α-Actin as a loading control). Right, quantitation of three independent repeats, *P* > 0.05, *t* test. (**G**) Normalized counts of GluClα peptides analysis (MS) of GluClα protein levels in postsynaptic densities extracted from GluClα-unedited and *wt* flies. *n* = 3 *P* > 0.05 paired *t* test. ns., not significant.

GluClα is an inhibitory channel in invertebrates that belongs to the Cys-loop ligand-gated family of ion channels (*38*), consisting of five homologous subunits that are arranged in a radial manner (Fig. 1A). Each subunit has an N-terminal extracellular hydrophilic domain containing ligand-binding residues, four transmembrane helices, and an intracellular long segment (*39*). Glutamate binding triggers a rapid influx of chloride ions, leading to hyperpolarization of the target cell (*38*, *40*). *Drosophila* GluClα is broadly expressed in the nervous system (*41*, *42*) and was shown in recent studies to control homeostatic modulation of presynaptic release at the neuromuscular junction (*43*), to regulate visual responses (*44*, *45*) and the processing of olfactory information (*40*). Here, we show that recoding of Ile^27^ to Val is necessary for proper olfactory responses, since flies expressing endogenous GluClα in which recoding of Ile^27^ is prevented exhibit reduced responses to appetitive and aversive odors and impaired pheromone-based social and sexual responses. The behavioral phenotypes of GluClα-unedited flies are associated with altered activity of olfactory projection neurons (PNs) specifically in the VA1v glomeruli and can be rescued by the expression of a fully edited form of GluClα in PNs. Our results demonstrate the physiological relevance of evolutionary conserved editing events in regulating complex behavior in *Drosophila* and may contribute to understanding the biophysical regulation of Cys-loop receptor-channels.

## RESULTS

### The recoding of Ile^27^ to Val in GluClα is evolutionarily conserved and exhibits high editing levels

A systematic comparison of conserved RNA editing events across six different *Drosophila* species identified a subset of highly conserved editing sites, suggestive of their functional importance (*46*). One of the conserved sites is found in the inhibitory glutamate-gated chloride channel (*GluCl*α). The transcript of *GluCl*α has six A-to-I editing sites, five of which are classified as nonsynonymous (Fig. 1A). Comparing the editing pattern of *GluCl*α among the six *Drosophila* species indicated that the recoding event of Ile^27^ located within the extracellular domain of the channel is conserved in *D. melanogaster*, *yakuba*, *simulans*, and *pseudoobscura* with more than 75% of the transcripts in all four species are edited (Fig. 1B and table S1). Next, we analyzed the spatial distribution of the six editing events in *GluCl*α across the fly brain using a dataset that we previously generated, containing transcriptomes of nine different neuronal populations including the relative editing levels of thousands of editing sites (*47*). We found that Ile^27^ to Val is the most highly edited site in all tested neuronal populations, ranging from 80 to 94% editing (Fig. 1C and table S2, A and B).

The three-dimensional (3D) structure of the *Drosophila* GluClα channel is not known. Yet, important structural information can be derived on the basis of homology with other Cys-loop receptors. The Ile to Val editing site is situated at position 27 of the immature protein. To locate the editing site in respect to the signal peptide, we have analyzed the full-length sequence of the *Drosophila* GluClα subunit using SignalP 6.0 (*48*). SignalP 6.0 predicted, with very high probability, a signal peptide that includes amino acids 1 to 22 (fig. S1A). Hence, the edited Ile^27^ is probably the fifth amino acid of the mature protein. We then submitted the full-length amino acid sequence of the immature protein to a secondary-structure prediction by PSIPRED 4.0 (*49*) and transmembrane topology prediction (*50*) in the PSIPRED server (*51*). These predictions support the location of Ile^27^ C-terminally (downstream) to the signal peptide and suggest that I27 is located N-terminally to a putative α helix of the *Drosophila* GluClα protein (fig. S1B). Very similar secondary-structure prediction obtained with JPred4 (*52*) corroborated the prediction by PSIPRED 4.0 (not shown). Multiple sequence alignment between the *Drosophila* GluClα subunit and various subunits of anionic Cys-loop receptors [performed by Clustal Omega (*53*)] indicates that the predicted α helix is aligned with the first α helix (termed α1) of Cys-loop receptor subunits whose 3D structure was determined at high resolution (fig. S1C). In addition, it reveals that the editing site (I27 to V) is indeed located very close to the N terminus of the mature protein, N-terminally to the first α helix (fig. S1C). However, this position is not included in the 3D structures of the various Cys-loop receptor subunits used for the multiple alignment (fig. S1C). Noteworthy in all known 3D structures of eukaryotic Cys-loop receptors, the N-terminal α helix (α1) proceeds by a loop that connects to the first β strand (β1). This loop contains a highly conserved amino-acid cluster (fig. S1C).

The high ratio of *GluCl*α transcripts that undergo editing at this particular site and its evolutionary conservation prompted us to further examine its functional relevance. To this end, we used the CRISPR/Cas9 system to impair editing at this site by removing the specific ECS (intronic region between positions 15,578,744 and 15,579,171) required for the binding of ADAR (Fig. 1D and fig. S2A). Comparing the editing pattern of all six sites between *GluCl*α*^unedited^* and matched genetic control showed that the deletion of this particular ECS abolished editing of Ile^27^ to Val and that of a close-by synonymous site that share the same ECS (Lys^26^ to Lys) while leaving the editing levels of the other sites intact (Fig. 1E and table S3). The removal of the ECS did not affect protein expression, as can be seen by comparable levels of GluClα protein in protein extracts from *GluClα^unedited^* and control flies (Fig. 1F). Taking into account the close proximity of Ile^27^ to the signal peptide, we verified that eliminating the recoding of Ile^27^ to Val did not impair the proper localization of the channel to the cell membrane. To this end, we isolated synaptosomes and postsynaptic densities from *wt* and *GluCl*α*^unedited^* flies (*54*) and compared the relative amount of GluClα protein using quantitative mass spectrometry (MS) analysis (Fig. 1G and table S4). Last, we confirmed that the elimination of editing at this site did not result in reduced life span and found that *GluCl*α*^unedited^* flies rather have an extended life span in comparison to *wt* controls (fig. S2B).

### Recoding of Ile^27^ to Val is necessary for proper olfactory responses

**Fig. 2.**
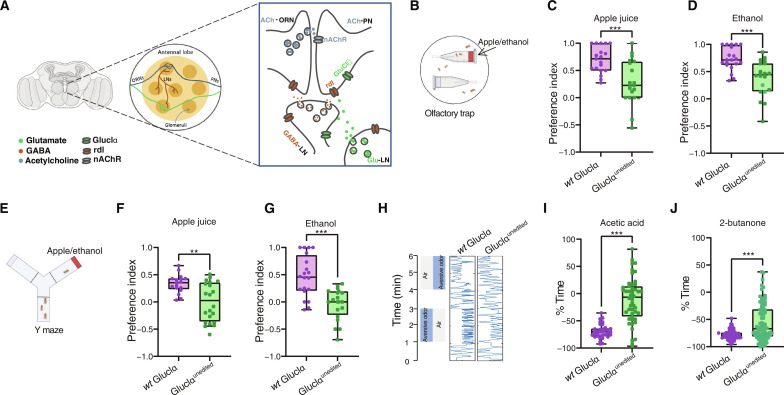
Ile^27^ recoding is necessary for proper olfactory responses. (**A**) Schematic illustration of GluClα function within the AL. PNs receive input from ORNs, and their response is dampened by glutamate that is released from LNs and acts on GluClα receptors. (**B**) Illustration of the two-choice olfactory trap paradigm, where flies are given a choice to enter a trap containing an odor compound (red agar) or without odor (clear agar), and the number of trapped flies is analyzed after 24 hours. Preference of GluClα^unedited^ and *wt* males toward apple juice (**C**) and ethanol (**D**) containing, *n* = 20 ***P < 0.0001; Mann-Whitney test. (**E**) Graphical depiction of Y-maze assay in which flies can climb toward odors placed at the end of the arms, and their amount on each side is used to calculate preference toward apple juice or ethanol. Preference of GluClα^unedited^ and *wt* males toward apple juice (**F**) or ethanol (**G**) as analyzed by the Y-maze assay. Unedited flies exhibit reduced attraction to apple juice and ethanol **P < 0.01; *T* test, *n* = 20. *wt* flies exhibit preference to apple juice and ethanol that differs significantly than 0 (one-sample *t* test; *P* < 0.0001), whereas GluClα^unedited^ do not differ than 0. (**H**) Schematics of the multiplex system that measures the fraction of time during which single flies move toward or away from acetic acid (**I**) and 2-butanone (**J**). Mean time of *wt* flies away from acetic differs or 2-butanone differs from zero [one sample *t* test; *P* value (two tailed) <0.0001], while unedited is no different from 0. Unedited flies show decreased aversion to acetic acid, *n* = 35 ***P < 0.0001; Mann-Whitney test, and toward 2-butanone, *n* = 40 ***P < 0.0001; Mann-Whitney test.

Given the established role of *GluCl*α in processing olfactory information (*40*), we tested the behavioral responses of *GluCl*α*^unedited^* flies to appetitive and aversive odors. Flies detect odors using olfactory receptor neurons (ORNs) located at the antennae and maxillary palps. In general, each ORN expresses a single odorant receptor gene (*55*–*58*). ORNs expressing the same receptor send their axons to the same glomerulus in the antennal lobe (AL) (*59*–*61*). In addition, the AL also contains second-order PNs, which receive input from a single glomerulus (*40*, *62*) and are responsible for delivering odor information to higher brain regions (*40*, *62*). The AL also contains local neurons (LNs). Most LNs are inhibitory GABAergic neurons, but about one-third of LNs are inhibitory glutamatergic neurons (*40*), which inhibit via *GluCl*α both PNs and LNs (Fig. 2A) (*40*).

Since attraction or aversion to odors requires intact motor capabilities, we first verified that *GluCl*α*^unedited^* flies exhibit normal locomotor behaviors by analyzing various features of their motor actions using the FlyBowl tracking–based behavioral analysis system (fig. S3 and table S7). Fruit flies use odors to navigate across complex environments and locate food sources (*63*). Ethanol and amine volatiles emitted from rotting fruits attract *Drosophila* flies, who aggregate on fermenting fruits as a hub for feeding, mating, and egg laying (*64*–*66*). To examine the attraction of *GluCl*α*^unedited^* flies to appetitive odors, we used the simple two-choice olfactory trap assay, in which one of the traps contained an appetitive odor such as apple juice or ethanol, whereas the other trap contained plan agar as control (Fig. 2B). Fifty 3-day-old flies were introduced into chambers containing two Eppendorf-based traps with either 1% agar or agar supplemented with 10% apple juice and were left uninterrupted for 24 hours before the number of flies in each trap was counted. Comparing the preference of *wt* flies to that of *GluCl*α*^unedited^* flies, we found that while *wt* flies exhibited strong preference toward apple juice, *GluClα^unedited^* flies showed a marked reduction in the number of flies that entered the apple juice traps (Fig. 2C). The reduced preference toward appetitive odor was also apparent toward ethanol (Fig. 2D). Next, we tested the attraction toward each of these odors using a simple Y-maze assay, in which flies can choose an arm containing at its end agar or agar with 10% apple juice or ethanol (Fig. 2E). Unlike the olfactory trap assay that measures the number of flies trapped in each of the traps over 24 hours, the Y maze is based on an immediate choice between the arms of the maze. Fifty 3-day-old *GluCl*α*^unedited^* or *wt* flies were inserted at the entrance of each Y maze apparatus, and the flies were given 40 s to choose between the two arms before the vials were cupped. While *wt* flies show a strong preference to apple juice and ethanol-containing arms, the mean preference of *GluCl*α*^unedited^* flies is close to zero (Fig. 2, F and G), implying a random choice between the two arms. The decreased attraction of *GluClα^unedited^* flies to apple juice and ethanol in two independent assays suggests that editing of Ile^27^ to Val is necessary for proper olfactory responses toward appetitive odors.

Next, we analyzed the response of *GluClα^unedited^* flies to aversive odors using the multiplex system (*67*). This single fly assay tracks temporal approaches toward an odor or avoidance from it and hence overcome the inherent limitation of olfactory trap and Y-maze systems that measure behavioral choices of groups in which the choice of individuals may be influenced by those of other flies (Fig. 2H). We used the multiplex system to examine the responses of *GluClα^unedited^* flies to two aversive odors: acetic acid and 2-butanone. Examining the time spent avoiding each of the odors showed that while the control (*wt*) flies had a negative value of close to −100, in agreement with robust avoidance, the average time depicted by GluClα^unedited^ flies was close to zero, suggesting that their movement within the chamber is random (Fig. 2, I and J). These results demonstrate that editing of Ile^27^ to Val in GluClα is necessary for proper chemotaxis.

### Recoding of Ile^27^ to Val is necessary for the expression of complex olfactory guided behaviors

**Fig. 3.**
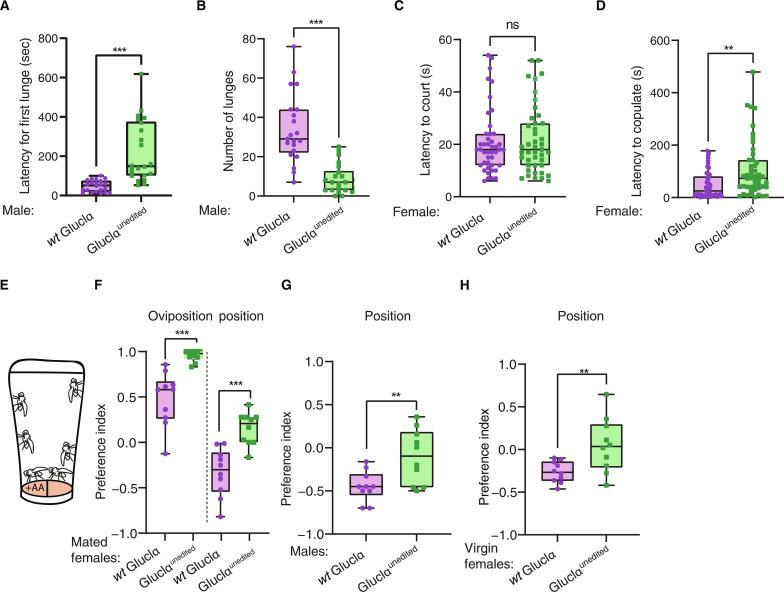
GluClα^unedited^ flies exhibit impaired olfactory-based behaviors. (**A**) GluClα^unedited^ flies exhibit longer duration to first lunge during aggression assay. *n* = 23, ***P < 0.0001; Mann-Whitney test. (**B**) GluClα^unedited^ flies exhibit a reduced ratio of lunges. *n* = 23, ***P < 0.0001; Mann-Whitney test. (**C**) *wt* male flies exhibit similar courtship behavior toward *wt* and GluClα-unedited female flies, manifested by similar latency to first courtship action. *n* = 50 *P* > 0.05 Mann-Whitney test. (**D**) GluClα^unedited^ female flies exhibit reduced receptivity to courtship by *wt* male flies as measured by the latency to mate. Take a similar amount of time to start courting mutant and *wt* cs females. *n* = 50, **P < 0.01; Mann-Whitney test. (**E**) Schematic illustration of two choice ovipositional and positional preference in egg laying on substrate with acetic acid. (**F**) Oviposition and position indices of mated GluClα^unedited^ and *wt* females. GluClα-unedited female exhibit enhanced oviposition index *n* = 10 (***P < 0.0001 one sample *t* test) and reduced positional aversion to acetic acid (**P < 0.005, *t* test, two-tailed), which significantly differ from zero (*P < 0.05, two-tailed *t* test). (**G**) GluClα^unedited^ males show reduced positional aversion to substrate containing acetic acid *n* = 10 (test). (**H**) Virgin GluClα^unedited^ female exhibit reduced positional aversion to substrate containing acetic acid *n* = 10 (test).

**Fig. 4.**
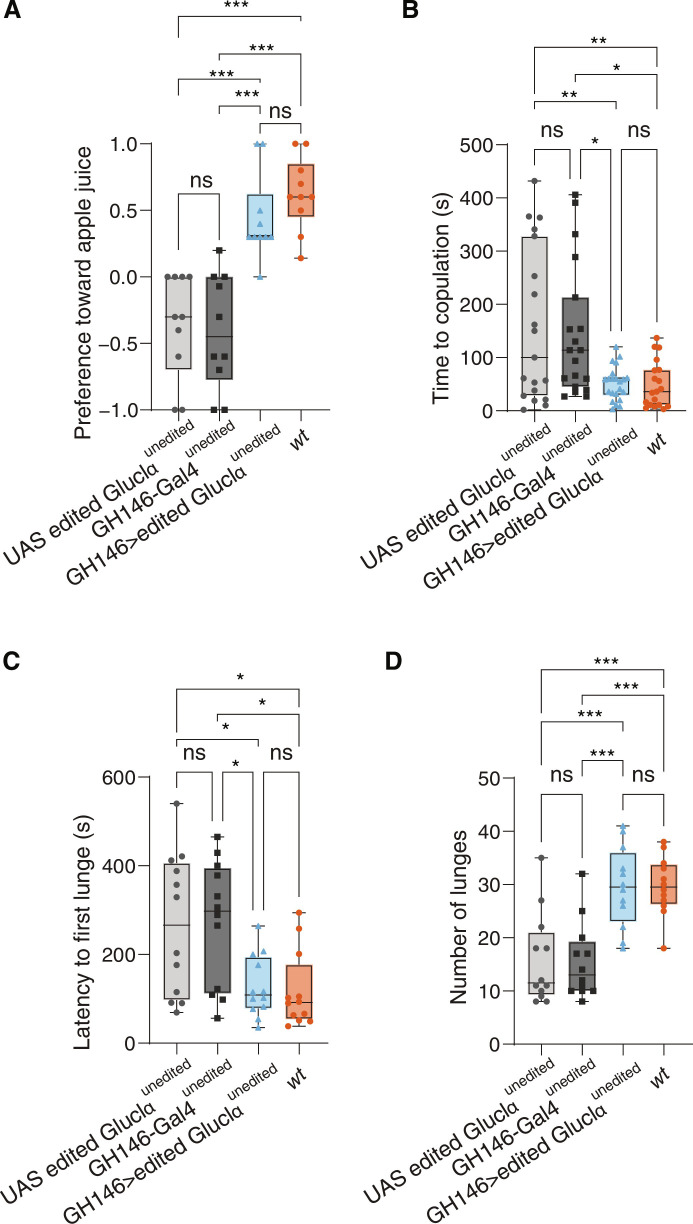
Expression of an edited channel in PNs restores proper olfactory responses. (**A**) Flies expressing an edited channel in PNs (blue) show increased preference toward apple juice, which is similar in its extent to *wt* controls (orange), compared to genetic controls (gray tones). *n* = 10, ***P < 0.0001; one-way analysis of variance (ANOVA). (**B**) Females expressing an edited channel in PNs exhibit similar receptivity to mate as *wt* controls, which is significantly higher than that of genetic controls. *n* = 20 **P < 0.001; one-way ANOVA. Males expressing an edited channel in PNs show shorter latency to first lunge (**C**), and an increase in the number of lunges (**D**) compared to the control flies. *n* = 12, *P < 0.005, ***P < 0.0001; one-way ANOVA.

Given the reduced response to appetitive and aversive odors, we next extended the analysis of GluClα^unedited^ flies to include behaviors that rely on olfactory cues, such as pheromone-based social interactions (*68*). A central pheromone in this respect is the male-specific pheromone cVA that is known to induce male-male aggression and promote sexual receptivity in female flies (*69*). Comparing aggression levels between pairs of GluClα^unedited^ flies to those of *wt* flies revealed a dramatic reduction in the number of lunges exhibited by GluClα^unedited^ flies (Fig. 3A) as well as longer duration until they exhibited first lunge (latency to first lunge, Fig. 3B). The overall reduction in aggressive behavior suggests impaired perception of cVA. These notable results prompt us to examine another cVA-dependent behavior in female flies, where the presence of cVA emitted from courting male flies promotes receptivity (*69*–*71*), measured by the time it takes from first courtship action until copulation (latency to copulation). For that, we paired GluClα^unedited^ or *wt* female flies with *wt* male flies and assayed their receptivity. First, we verified that *wt* male flies exhibit similar courtship patterns toward GluClα^unedited^ and *wt* females and found that the time it took male flies to initiate courtship action was similar toward the two genotypes (Fig. 3C). Next, we measured the time it took from initiation of courtship action by male flies and until the beginning of copulation. GluClα^unedited^ females exhibited significantly longer latency to copulate, which reflects reduced receptivity (Fig. 4D). The reduced receptivity agrees with impaired perception of cVA.

We then explored the phenotype GluClα^unedited^ female flies in a two-choice egg-laying paradigm, in which females exhibit attraction to food containing acetic acid as an egg-laying substrate, and at the same time show aversion to staying on acetic acid–containing food (positional avoidance) (*72*). The oviposition attraction to acetic acid is mediated by gustatory neurons and the positional aversion by olfactory cues (*72*, *73*). The trade-off between oviposition preference and positional avoidance is an intriguing system to test the behavior of GluClα^unedited^ female flies. The experimental design consists of a simple apparatus in which mated females are allowed the choice to lay eggs on regular food or on food containing 5% acetic acid, and the number of eggs laid on both sides is counted after 3 hours. During this period, the position of females is monitored every 15 min to calculate positional preference/aversion toward acetic acid–containing substrate (Fig. 3E). As expected, *wt* female flies show a robust preference to lay eggs on food with acetic acid (positive oviposition preference values), while they avoid staying there (negative positional preference values, Fig. 3F), demonstrating the opposing motivations between the need to lay eggs in a substrate containing acetic acid and their avoidance from its aversive odor. GluClα^unedited^ females, on the other hand, lost the positional aversion to acetic acid and instead showed a slight preference to be on acetic acid food and accordingly showed a significantly higher preference to lay all their eggs on this substrate (Fig. 3F). Moreover, GluClα^unedited^ males and virgin female that lack the motivation to lay eggs, and therefore do not need to stay on acetic acid as a substrate for egg laying, do not show positional aversion to acetic acid, and spend on average equal time on both substrates (Fig. 3, G and H). The results suggest that while the gustatory perception of acetic acid that is responsible for oviposition is normal in GluClα^unedited^ females, their olfactory perception of acetic acid is impaired, pointing to the importance of Ile^27^ recoding in the processing of olfactory information.

### The recoding of Ile^27^ to Val is necessary in PNs for the proper expression of olfactory-based behaviors

Considering the olfactory-related phenotypes observed in GluClα^unedited^ flies, and the fact that these flies express an unedited form of GluClα in all the cells that normally express GluClα, we next searched for the neurons in which the recoding of Ile^27^ to Val has a functional relevance. Since GluClα was previously shown to function in olfactory PNs (*40*), we tested whether expressing a fully edited version of GluClα in olfactory PNs of GluClα^unedited^ flies can rescue the olfactory phenotypes. To this end, we generated GluClα^unedited^ flies harboring the GH146 driver that target ~90 of the 200 PNs in the AL (*74*) and a UAS transgene that allows the expression of GluClα protein in which the six editing sites are in a fully edited state (the A nucleotide is mutated to G). We assumed that introducing an excess of edited channels can restore the editing level of this particular site to normal levels. Pan neuronal expression of the fully edited channel in GluClα^unedited^ flies resulted in 90% of the *GluCl*α transcripts harboring a G nucleotide (edited version) at this position (fig. S4A).

We next profiled the behavior of GluClα^unedited^ flies expressing a fully edited version of GluClα in PNs using four behavioral paradigms. First, we used the FlyBowl system to make sure that the overexpression of the edited channel did not result in any motor defects (figs. S4B and tables S6 and S7). Next, we examined their attraction to apple juice using an olfactory trap assay. While both genetic controls exhibit no preference for apple juice, the expression of edited GluClα in PNs led to a clear preference toward apple juice (similar in its extent to *wt* controls), suggesting that the expression edited version can rescue the impairment in olfactory response to appetitive odors (Fig. 4A). Furthermore, GluClα^unedited^ females harboring the edited form in PNs, exhibited a significant increase in their receptivity to courting males, as the latency to copulation was dramatically shorter than the genetic controls and was similar in its extent to *wt* female flies (Fig. 4B). Expressing the edited form in PNs also restored aggressive display, as the experimental males exhibited shorter duration to the expression of first lunge and higher number of lunges in comparison to genetic controls (Fig. 4, C and D). Together, our results propose that the loss-of-function phenotypes of GluClα^unedited^ flies can be partially restored by the expression of edited version in PNs, suggesting that the recoding of Ile^27^ to Val has a functional role in PNs to facilitate olfactory responses that are similar in their extent to wt flies.

### Recoding of I27V in GluClα shapes odor responses within the antennal lobe

**Fig. 5.**
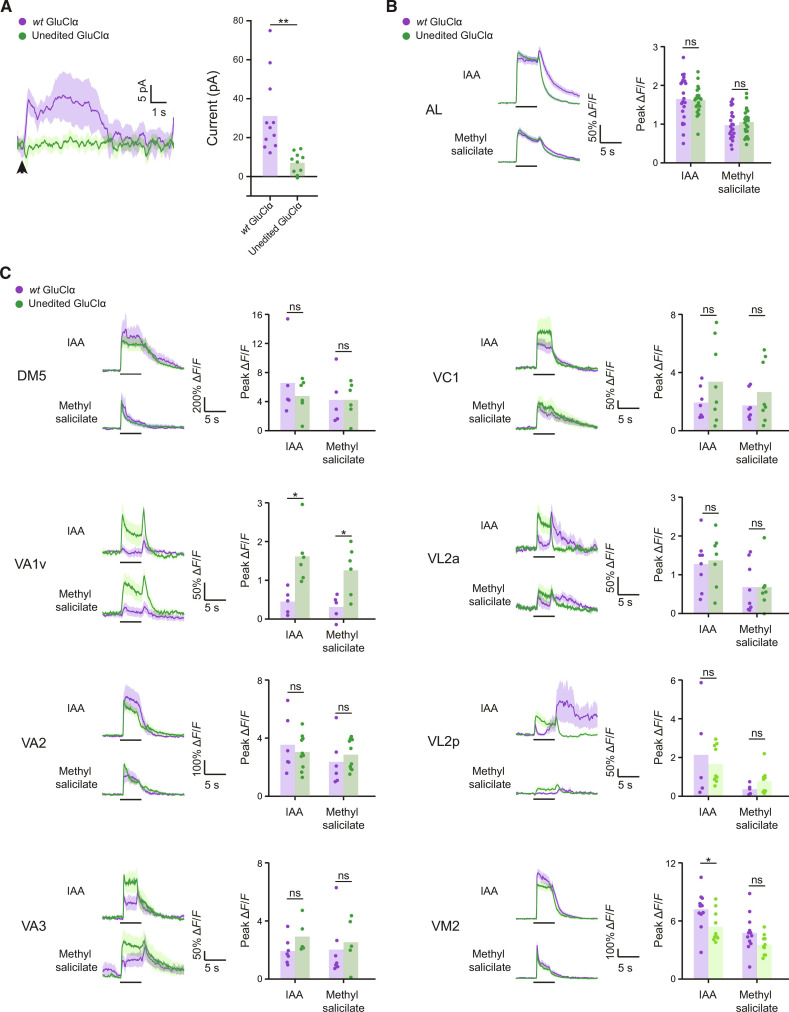
GluClα-editing state affects PN odor responses. (**A**) Left, inhibitory currents evoked by the application of 1 mM glutamate (arrowhead) in PNs in *wt* (purple) and GluClα^unedited^ (green). Data are means (solid line) ± SEM (shaded area). Right, analysis of maximal current of traces on the left. Each dot represents measurement from a single fly. **P < 0.01, *t* test, *n* = 11 and 10 for *wt* and GluClα^unedited^, respectively. (**B**) ∆*F*/*F* of GCaMP6f signal in the entire AL in control GluClα (purple) and unedited GluClα (green) flies during presentation of odor pulses (isoamyl acetate, IAA, and methyl salicylate, horizontal lines). Data are means (solid line) ± SEM (shaded area). No significant difference in odor responses was observed between flies with GluClα^unedited^ background to the control flies with *wt* background. Paired *t* test, *n* = 5. (**C**) ∆*F*/*F* of GCaMP6f signals in eight different glomeruli to IAA and methyl salicylate in GluClα^unedited^ flies (green) compared to *wt* flies (purple). A significant increase in odor response was observed in VA1v glomerulus for the two tested odors. *P value < 0.05; *t* test, *n* = 6.

It was previously shown that glutamate functions as an inhibitory transmitter that shapes PNs response to olfactory stimuli (*40*). Glutamate that is secreted from LNs, binds to GluClα on PNs and inhibits their activity, as knockdown of GluClα in PNs results in increased responses of certain glomeruli to isoamyl acetate (IAA) and methyl salicylate (*40*). Thus, we first examined the responses of GluClα^unedited^ and *wt* flies to glutamate. To this end, we used in vivo whole-cell patch clamp recording and measured glutamate-evoked currents in PN. GluClα^unedited^ flies showed a strong and significant reduction in glutamate-induced currents (Fig. 5A). To strengthen the functional role of the edited form of GluClα in PNs, we next explored its effect on neuronal physiology by analyzing the odor-evoked neuronal activity of PNs within the AL. To this end, we expressed GCaMP6m in PNs in GluClα^unedited^ and *wt* flies and performed two-photon functional Ca^2+^ imaging in response to IAA and methyl salicylate. We first validated that the GluClα^unedited^ flies harboring either the PN driver or the GCaMP6m exhibit reduced attraction to appetitive odor compared to *wt* flies that harbor the same transgenes (fig. S5). Next, we compared that odor-evoked activity across all glomeruli and found similar responses to both odors in GluClα^unedited^ and *wt* PNs (Fig. 5B). The overall similarity in the responses when examining all glomeruli prompted us to analyze the responses of single glomeruli across the AL including DM5, VA1v, VA2, VA3, VC1, VL2a, VL2p, and VM2. While most tested glomeruli exhibited similar responses in GluClα^unedited^ and *wt* flies, the VA1v glomerulus exhibited significantly increased activity in GluClα^unedited^ flies in response to IAA and methyl salicylate (Fig. 5C). The enhanced responses of VA1v PNs to IAA and methyl salicylate in unedited flies suggest that the recoding event of Ile^27^ in GluClα is necessary to dampen the activity of the PNs during the perception of these odors. We also observed a small reduction in the responses to IAA in the case of the VM2 glomerulus. These results support our behavioral finding, all together indicating that the recoding of Ile^27^ is required for correct function of AL neurons in regulating olfactory-based behavioral responses.

## DISCUSSION

A-to-I RNA editing has long been suggested as cellular machinery that can provide the proteomic diversity required for the intricate function of the nervous system by shaping the spatial and temporal repertoire of expressed protein isoforms expressed in neurons (*10*, *75*–*78*). Here, we dissected the physiological importance of an editing site in *Drosophila* and showed that a highly abundant and evolutionarily conserved recoding event that occurs within the extracellular domain of *GluCl*α is necessary for proper olfactory responses in adult flies. First, by ablating the editing of Ile^27^ to Val such that the endogenous channel harbors only the unedited version (Ile^27^ instead of Val), we were able to examine the contribution of this particular recoding event to its function in behaving animals. Second, by using a set of behavioral assays, we found that the edited isoform is necessary for proper perception of odors, as well as for the expression of pheromone-based social behaviors. Furthermore, we mapped the spatial requirement of the edited isoform to olfactory PNs. The behavioral phenotypes observed in the unedited flies do not stem from impaired health or motor defects as they exhibit intact motor activity and even extended lifespan. The last is in agreement with findings in *Caenorhabditis elegans* where mutations that cause defects in sensory neurons lead to extended life span (*79*, *80*). Moreover, the behavioral phenotypes of the unedited flies could be rescued by the expression of an edited channel in PNs, resulting in olfactory performance that is similar to that of *wt* flies.

Consistent with a previous study (*40*), our results indicate that the unedited isoform of *GluCl*α has reduced glutamate-evoked currents and affects odor responses in VA1v glomerulus. Considering results from previous studies that found that silencing one or a few glomeruli had no effect on behavioral output (*81*), it is unclear how the lack of *GluCl*α editing in PNs has such a pronounced effect on behavior. The affected glomerulus (VA1v) is sexually dimorphic (*82*–*84*) receiving input from Or47b neurons known to respond to conspecific odors. PNs responding to food odors are known to be less specific than their cognate ORNs (*85*, *86*). In *GluClα^unedited^* flies, the VA1v glomerulus loses its specificity and responds to non-pheromone odors, suggesting that under normal conditions, glutamatergic inhibition is required to maintain VA1v specificity. It is therefore possible that the loss of odor valence observed in unedited flies results from improper activation of pheromonal pathways during the perception of non-pheromonal odor signals, leading together to contradicting consequences. Whether this is indeed the case is out of the scope of this manuscript. Furthermore, it would be intriguing to examine in future work whether *GluCl*α has a role in controlling the activity in other pheromone related glomeruli.

The current case is not the first recoding event that is necessary for the proper function of a channel. The ablation of an editing site located within the mammalian excitatory ionotropic glutamate receptor subunit B leads to early seizure-related death in mice (*87*). While the ablation of Ile^27^ recoding in *GluCl*α did not reduce viability or reproduction of flies under laboratory conditions, the impaired olfactory responses exhibited by unedited flies are expected to affect their fitness in natural environments, since flies heavily rely on their olfactory system to survive and reproduce.

The loss-of-function phenotype of the unedited form indicates that recoding of Ile^27^ to Val is necessary for the proper function of the channel, and the combination of high editing levels and evolutionary conservation are useful criteria when predicting which of the many editing events has the potential of having a function. This brings up the question of why evolution chose to maintain modulation of the sequence at the RNA level rather than inserting a mutation at the DNA level. The answer probably lies in the varying levels of its editing between different neuronal populations, suggesting that the ratio between edited and unedited forms of the channel or within subunits that construct the same channel may be spatially regulated to fine-tune its function. A relevant example in this respect is the spatial and temporal regulation of a recoding event that is found within the calmodulin-binding IQ domain of the voltage-activated calcium channel (Ca_v_1.3) and how ablation enhances learning (*16*, *17*). Further studies are required to test whether the editing repertoire of *GluCl*α is regulated in response to different environmental conditions.

**Fig. 6.**
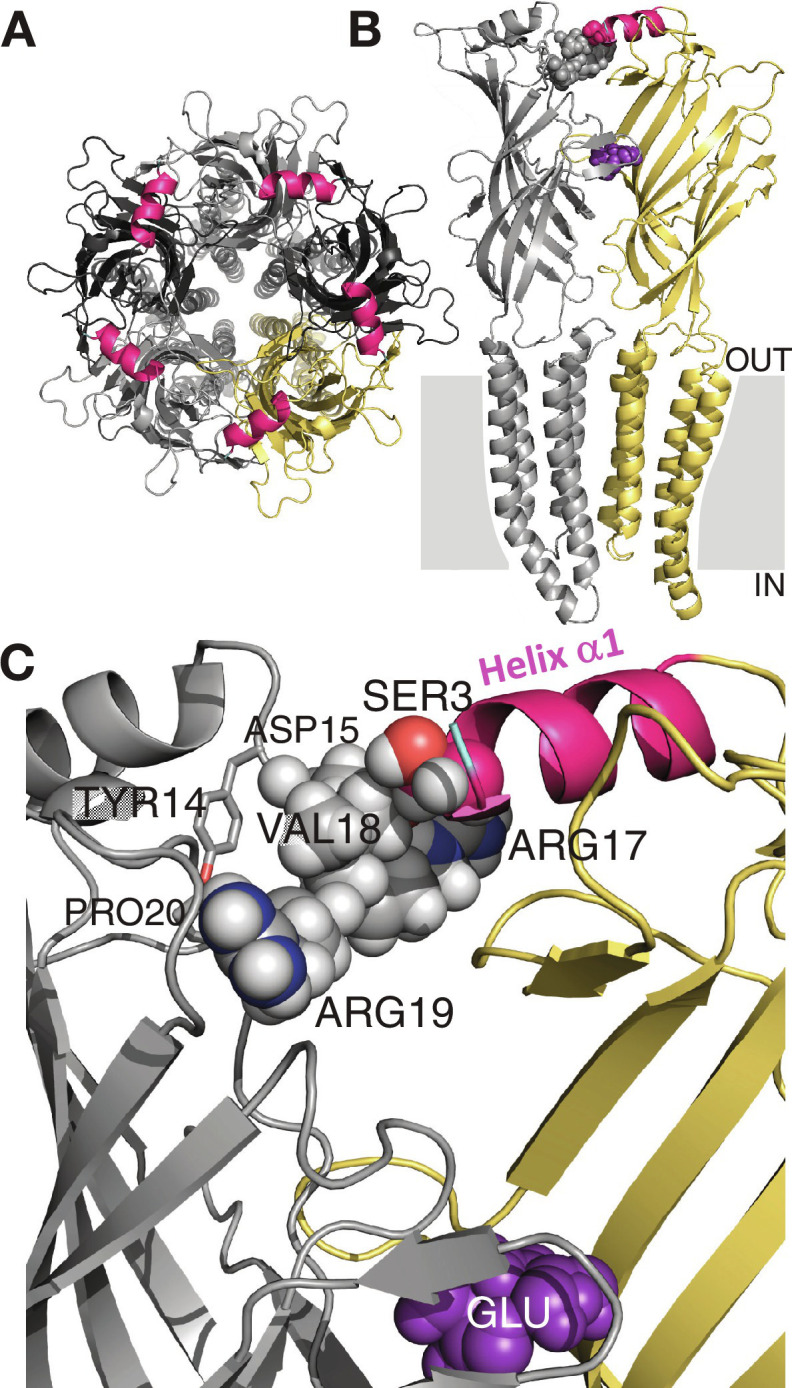
Structural characteristics of GluClα receptors. (**A**) Top view of the *Caenorhabditis elegans* GluClα receptor (GluClα_cryst_R; Protein Data Bank ID code 3RIF) showing five identical subunits, which are colored differently to highlight the intersubunit interfaces. Alpha-helix α1 is colored in pink in all five subunits. (**B**) Two adjacent subunits (colored in gray and yellow) of the GluClα_cryst_R are viewed from the side. The membrane region is illustrated as a gray rectangle interrupted by the transmembrane helices (OUT, extracellular; IN, intracellular). The N-terminal tip of a-helix α1 (pink), which belongs to the yellow-colored subunit, interacts with the adjacent subunit (gray spheres), as detailed in (C). The neurotransmitter glutamate (purple spheres) is bound at the intersubunit interface. (**C**) Space-filling models of the side chains of SER3 (belongs to Helix a1) and VAL18 (belongs to the conserved cluster shown in fig. S1) indicate van der Waals interactions between these two amino acids (3.9-Å center-to-center distance between O^γ^ of SER3 and C^γ2^ of VAL18). Side chains of the other amino acids belonging to the conserved cluster are shown as spheres or sticks. Carbon, oxygen, nitrogen, and hydrogen atoms are colored in pink or gray, red, blue, and white, respectively. Glutamate is shown in purple spheres.

It is intriguing that a recoding event that is conservative in its nature (Ile to Val) and that is located close to the N terminal tip of the channel (five amino acids downstream to the putative signal peptide) has such a strong impact on function. As of this moment, there is no resolved 3D structure of *Drosophila* GluClα, and 3D structures of other Cys-loop receptor-channels lacked a portion of the N terminal segment that contains this particular residue, making it difficult to assess the molecular function of Ile^27^ recoding to Val. Still, there are notable examples of the way by which a similar recoding event (Ile^400^ to Val) that is located at the intracellular cavity of the potassium channel K_v_1.1 strongly modifies the inactivation kinetics, where the edited form of the channel recovers from inactivation 20 times faster than the unedited form (*88*). The faster recovery is achieved because of reduced hydrophobic interaction between the tip of the inactivation gate and the Val residue that is found within the intracellular cavity (*88*). While the Ile^27^-containing segment of the *Drosophila* GluClα subunit is missing in the various 3D structures of anionic Cys-loop receptors (fig. S1), its location is clearly a few amino acids upstream to the N-terminal α helix (α1) that is typical of Cys-loop receptors (fig. S1). Helix α1 lies in close proximity to a conserved amino acid cluster that belongs to the adjacent subunit (e.g., Fig. 6, A to C; for cluster sequence, see fig. S1). This close proximity allows for van der Waals interactions (*89*) (Fig. 6C), and in some cases hydrogen bonding (*90*) [e.g., PDB 6PLR (*90*)], between entities of α1-helix and amino acids belonging to the aforementioned cluster in the adjacent subunit. One may therefore envision that in the *Drosophila* GluClα receptor the N terminus and the editing site itself might be close, at an interaction distance, to the neighboring subunit. Since inter-subunit interactions often take part in the function of Cys-loop receptors, further studies are necessary to assess how the recoding of this position affects the biophysical properties of the *Drosophila* GluClα receptor.

## MATERIALS AND METHODS

### Analyzing evolutionary conservation of GluClα editing sites across *Drosophila* species

The analysis is based on an existing dataset from Ramaswami *et al.* (*91*) that quantified RNA editing at 605 loci using a multiplex microfluidic polymerase chain reaction (PCR) with deep sequencing (mmPCR-seq) across 131 Drosophila strains (*n* = 2 per strain). The study used multiplex PCR primers to amplify 605 loci that include many known RNA editing events. PCR products of each sample were then subjected to a 15-cycle barcode PCR and pooled together. The library was sequenced using Illumina HiSeq with 101–base pair (bp) paired-end reads. Paired-end reads were combined and mapped onto the genome (dm3) using BWA samse allowing nine mismatches per read. The sequencing reads were aligned to a combination of the reference genome and 100-bp exonic sequences surrounding known splicing junctions from available gene models (obtained from the UCSC genome browser). We quantified editing levels of known *D. melanogaster* RNA editing sites by taking the fraction of reads containing a “G” nucleotide at that position. For editing level quantification, sites covered by ≥50 mmPCR-seq reads were used. For each strain, we excluded editing sites where the measured editing levels in the two biological replicates differed by >20% (see Fig. 1B). Custom scripts used to process data are available upon request. In this study, we compared the relative editing levels of six editing sites in GluClα.

### Analysis of GluClα editing levels across the fly brain

We used an existing dataset (*47*) and used the following analysis pipeline: STAR (v2.4.2) (1) (--twopassMode Basic) was used to map paired-end mmPCR-seq reads and single-end RNA sequencing reads to the dm6 genome as described above. We then used the Samtools mpileup function to determine base calls from uniquely mapped reads at known and previously unidentified editing sites and calculated editing levels as number of G reads divided by the total of both A and G reads at a site. For mmPCR-seq, we required each replicate to have 100× coverage and we removed sites that were not within 20% editing between replicates, as done previously (*91*). Final mmPCR editing levels were determined after down sampling coverage to 200 reads for statistical analysis.

### Fly lines

All *D. melanogaster* fly lines used in this study were kept at 25°C, ~50% humidity, light/dark of 12:12 hours, and maintained on cornmeal, yeast, molasses, and agar medium. Most fly lines were backcrossed to a Canton S background or, if mentioned, were maintained on w1118 background. *wt* GluClα and unedited GluClα were used for behavioral assays. The genotype of GluClα-unedited flies was validated in each experiment using PCR analysis (see below). GH146-Gal4 > UAS GCaMP6 harboring *wt* or unedited version of GluClα were used for the Ca^2+^ imaging experiments. UAS edited GluClα flies were a generous gift from L. Keegan.

### Generation of GluClα-unedited flies

Ablation of Ile^27^ editing site was achieved by deletion of the of intronic ECS region: chr3R: 15578744-15579171 using CRISPR-Cas9 and the following gRNAs to induce double-stranded cuts to the DNA, on each side of the ECS: TCTAAACCCTAGATATACGCTGG and TATAGTATGTGACTTTGCCTGGG. In addition, we made synonymous mutations to the sgRNA target region or protospacer adjacent motif to prevent recutting by the Cas9-CRISPR system. The genotype of the GluClα-unedited flies was validated by performing simple PCR analysis (fig. S2A) using the following primers: Forward: TTGTCTCCCGCTCCACTTAC. Reverse: TTGGGCAATTTGAAAGTCGAAA.

### Western blot analysis

Relative levels of GluClα was analyzed using Western blot analysis (20 heads) and anti-GluClα antibodies produced in rabbit (1:1000) and anti-actin as a loading control. Densitometric analysis was used to compare relative protein levels of GluClα unedited and *wt *channels. GluClα protein levels were normalized according to actin levels.

### Isolation of synaptosomes and postsynaptic densities

A protocol for the isolation of synaptosomes and postsynaptic densities as described by Rajkimar *et al.* (*54*) was adapted to using fly heads as a source for protein using the changes. *wt* and GluClα-unedited flies were flash frozen. Nine hundred heads of each were homogenized in 1 ml of buffer A (40 strikes in dounce homogenizer). The resulting postsynaptic density fraction was dissolved in 40 μl of buffer C. Five hundred and eighty-four micrograms of protein was separated in a 10% polyacrylamide gel and stained using Comassie staining. All bands at the size of 45 to 60 KDa were sent for MS analysis.

### In-gel proteolysis and MS analysis

The proteins in the gel slices were reduced with 3 mM dithiothreitol (60°C for 30 min), modified with 10 mM iodoacetamide in 100 mM ammonium bicarbonate (in the dark, room temperature for 30 min), and digested in 10% acetonitrile and 10 mM ammonium bicarbonate with modified trypsin (Promega) at a 1:10 enzyme-to-substrate ratio, overnight at 37°C. The tryptic peptides were desalted using C18 tips (Homemade stage tips), dried, and resuspended in 0.1% formic acid. The tryptic peptides were desalted using C18 tips (Top tip, Glygen), dried, and resuspended in 0.1% formic acid. The peptides were resolved by reverse-phase chromatography on 0.075 × 180-mm fused silica capillaries (J&W) packed with Reprosil reversed-phase material (Dr Maisch GmbH, Germany). The peptides were eluted with different concentration of acetonitrile with 0.1% of formic acid: a linear 60-min gradient of 6 to 34% of 80% acetonitrile followed by a 15-min gradient of 34 to 95% and 15 min at 95% of 80% acetonitrile with 0.1% formic acid in water at flow rates of 0.15 μl/min. MS was performed by Q Exactive HF mass spectrometer (Thermo Fisher Scientific) in a positive mode (*m*/*z* 300 to 1500, resolution 60,000 for MS1 and 15,000 for MS2) using repetitively full MS scan followed by higher-energy collisional dissociation (HCD; at 27 normalized collision energy) of the 18 most dominant ions (>1 charges) selected from the first MS scan. A dynamic exclusion list was enabled with exclusion duration of 20 s. The MS data were analyzed using the MaxQuant software 2.1.3.0 (*92*) for peak picking and identification using the Andromeda search engine, searching against the *D. melanogaster* proteome from the UniProt database with mass tolerance of 6 parts per million (ppm) for the precursor masses and 20 ppm for the fragment ions. Oxidation on methionine and protein N terminus acetylation were accepted as variable modifications, and carbamidomethyl on cysteine was accepted as static modifications. Minimal peptide length was set to six amino acids, and a maximum of two miscleavages was allowed. The data were quantified by label-free analysis using the same software. Peptide- and protein-level false discovery rates (FDRs) were filtered to 1% using the target-decoy strategy. Protein table were filtered to eliminate the identifications from the reverse database and common contaminants and single peptide identifications.

### Life span

*wt* and GluClα-unedited flies (100 of each) were collected upon eclosion and placed in single food vials. The number of live flies was recorded 3 days. Flies were transferred to fresh vials once a week. Log-rank test (REF) with FDR correction was performed to compare the survival curves.

### Determining relative editing levels using Sanger sequencing

GluClα-unedited flies expressing a fully edited channel in all neurons (Elav>GluClα edited) and genetic controls were flash frozen (*n* = 3 independent repeats). Total RNA was extracted from heads using TRIzol reagent (Ambion). One microgram of RNA was converted to cDNA using ProtoScript II reverse transcriptase, and sequences around I27V in GluClα transcripts were amplified using PCR (40 cycles) using the following primers: Forward primer: TCTTATACTTTGCCAGCCTGT. Reverse primer: TGTTCACGGAAGGTTAACTGC. PCR products were cleaned using EPPiC Fast and sent for Sanger sequencing.

### Behavioral experiments

All behavioral experiments were performed about an hour after the lights on.

#### FlyBowl

Male flies were collected upon eclosion and aged in groups of 10 flies per vial for 4 days before test. Ten flies of each genotype were inserted in groups of 10 into Fly Bowl arenas (*93*), and their behavior was recorded for 30 min and analyzed using CTRAX, FixTrax (*94*), and JAABA (*93*). For kinetic features, scripts were written in MATLAB to use the JAABA code to generate the statistical features as specified in Kabra *et al.* (*93*). Quantification of complex behaviors was done using JAABA Classifiers (*93*) to identify specific behaviors: walk, stop, turn, approach, touch, chase, chain, song, social clustering, and grooming. Each feature of the Fly Bowl experiment was standardized according to all values calculated in our experiments for that feature to generate a *z*-score. Scatter plots were created using R.

#### Olfactory trap assay

Traps consisting of an Eppendorf tube connected to a 200-μl pipette tip were fitted to the trap to prevent flies from being able to escape the odor trap after entering it. Each trap is filled with 500 μl of a 1% agar supplemented with 10% odor apple juice (*66*), ethanol, or water as a control. Each setup contains two traps (with odor and control) that are glued to a 90 mm–by–15 mm petri dish. The two traps are placed in opposite directions. Fifty 3-day-old flies are introduced into each such experimental system. The flies can be attracted to one of the traps based on smell alone since they cannot make physical contact with the substrate to make the decision. To increase the motivation to choose one of the traps, the test was done in the absence of food. The experimental system is wrapped in aluminum foil to prevent the penetration of light that could disrupt their decision to enter one of the traps based solely on smell. The number of flies in each trap is counted after 24 hours to calculate preference index: preference index (PI) = (# of flies that entered the trap with the tested odor − # of flies that entered the trap that does not contain an odor source)/(total # of flies).

#### Immediate odor preference using Y maze

The behavioral setup is composed of two vials containing agar with and without odors that are connected to the Y-shaped adapter. A vial containing 50 3-day-old flies is placed at the entrance of the adapter, and the flies are allowed to choose one of the arms. After 40 s, the upper vials are closed and the number of flies in each is counted. Preference index is calculated as follows: PI = (# of flies in test arm − # of flies in control arm)/(total # of flies).

#### Single-fly assay for odor preference

Behavioral experiments were performed in a custom-built, fully automated apparatus as described in detail (*67*). Single flies were introduced into clear polycarbonate chambers (length, 50 mm; width, 5 mm; height, 1.3 mm) and were exposed to air containing odor flow. Odors were prepared at 10-fold dilution in mineral oil. Fresh odors were prepared daily. Air or odor streams from the two halves of the chamber converged at a central choice zone. The location of the flies was recorded and tracked. A fly’s preference was calculated as the percentage of time that it spent on one side of the chamber.

#### Two-choice oviposition and positional assays

The two-choice egg-laying setup contains a standard fly bottle, with the base cut off and replaced with a 60-mm petri dish lid. Half of the petri dish lid contains solid food substrate with 5% acetic acid, and the other half contains food supplemented with equal volume of water. Two-choice dishes were made by dividing a 35-mm petri dish lid with a razor blade and pouring two samples of food substrate into each half. For each test, 15 to 20 recently mated females were introduced into each two-choice apparatus allowed to sample and lay eggs for 3 hours. Oviposition preference was determined by counting the number of eggs on each half of the two-choice dish (oviposition index = # of eggs laid on acetic acid–containing food − # of eggs laid on control food/# of total eggs laid). For positional preference, the number of flies on each half of the dish was counted every 15 min along the entire duration of the experiment (3 hours) (position index = average # of flies on acetic acid half − average # of flies on control food half/total # of flies).

#### Courtship and mating tests

Male flies were collected upon eclosion and aged in groups of 10 flies per vial for 4 days before test. Courtship arenas were placed in behavior chambers, under controlled temperature and humidity (25°C, 70% humidity). Behavior was recorded for 1 hour from the introduction of male and female pairs using Point-Grey Flea3 cameras (1080 × 720 pixels at 30 fps). Latency to copulate was quantified for each pair as total time, starting from the first wing vibration the male exhibited and ending in successful copulation.

#### Aggression

Four-day-old pairs of single-housed male flies (*wt*, GluClα, or when indicated GH146 > UAS edited GluClα and genetic controls) were put into round aggression arenas (about 0.08 cm^3^ in volume). A mixture of agarose and apple juice (1% agarose, 50% apple juice) was inserted into arenas to enhance aggressive behavior. Experiments were performed at a similar time of day (lights ON +1 hour). The flies’ behavior was recorded for 30 min with Point-Grey Flea3 (1080 × 720 pixels at 60 fps). Aggressive behavior was later quantified by counting the number of lunges for each pair and latency as the time from start of experiment to first lunge for each pair.

#### Functional imaging

Flies used for functional imaging were raised on cornmeal agar under a 12-hour light/12-hour dark cycle at 25°C. Imaging was done as previously described (*95*–*99*). Briefly, the flies were anesthetized on ice, and then a single fly was moved to a custom-built chamber and fixed to aluminum foil using wax. Cuticle and trachea in the required area were removed, and the exposed brain was superfused with carbonated solution as described above. Odors (purest level available) were obtained from Sigma-Aldrich (Rehovot, Israel). Odor flow of 0.4 l/min (10 to 1 dilution) was combined with a carrier air stream of 0.4 l/min using mass-flow controllers (Sensirion) and software-controlled solenoid valves (The Lee Company). This resulted in a final odor dilution of 5 × 10^−2^ delivered to the fly. Odor flow was delivered through a 1/16-inch ultrachemical-resistant Versilon PVC tubing (Saint-Gobain, NJ, USA) placed 5 mm from the fly’s antenna. Functional imaging was performed using a two-photon laser-scanning microscope (DF-Scope installed on an Olympus BX51WI microscope). Fluorescence was excited by a Ti-Sapphire laser (Mai Tai HP DS, 100-fs pulses) centered at 910 nm, attenuated by a Pockels cell (Conoptics), and coupled to a galvo-resonant scanner. Excitation light was focused by a 20×, 1.0–numerical aperture objective (Olympus XLUMPLFLN20XW), and emitted photons were detected by GaAsP photomultiplier tubes (Hamamatsu Photonics, H10770PA-40SEL), whose currents were amplified (Hamamatsu HC-130-INV) and transferred to the imaging computer (MScan 2.3.01). All imaging experiments were acquired at 30 Hz.

#### Glutamate administration

Glutamate (Sigma-Aldrich, G1251) solution was prepared and diluted in external solution to the final concentration of 1 mM. A glass pipette filled with glutamate solution was placed in close proximity to the AL and was emptied using a pico injector (Harvard Apparatus, PLI-100).

## References

[1] E. Eisenberg, E. Y. Levanon, A-to-I RNA editing–immune protector and transcriptome diversifier. Nat. Rev. Genet. 19, 473–490 (2018). 29692414 10.1038/s41576-018-0006-1

[2] B. L. Bass, RNA editing by adenosine deaminases that act on RNA. Annu. Rev. Biochem. 71, 817–846 (2002). 12045112 10.1146/annurev.biochem.71.110601.135501 PMC1823043

[3] S. H. Roth, E. Y. Levanon, E. Eisenberg, Genome-wide quantification of ADAR adenosine-to-inosine RNA editing activity. Nat. Methods 16, 1131–1138 (2019). 31636457 10.1038/s41592-019-0610-9

[4] P. Reautschnig, N. Wahn, J. Wettengel, A. E. Schulz, N. Latifi, P. Vogel, T.-W. Kang, L. S. Pfeiffer, C. Zarges, U. Naumann, L. Zender, J. B. Li, T. Stafforst, CLUSTER guide RNAs enable precise and efficient RNA editing with endogenous ADAR enzymes in vivo. Nat. Biotechnol. 40, 759–768 (2022). 34980913 10.1038/s41587-021-01105-0

[5] C. X. George, G. Ramaswami, J. B. Li, C. E. Samuel, Editing of cellular Self-RNAs by adenosine deaminase ADAR1 suppresses innate immune stress responses. J. Biol. Chem. 291, 6158–6168 (2016). 26817845 10.1074/jbc.M115.709014 PMC4813567

[6] B. J. Liddicoat, R. Piskol, A. M. Chalk, G. Ramaswami, M. Higuchi, J. C. Hartner, J. B. Li, P. H. Seeburg, C. R. Walkley, RNA editing by ADAR1 prevents MDA5 sensing of endogenous dsRNA as nonself. Science 349, 1115–1120 (2015). 26275108 10.1126/science.aac7049 PMC5444807

[7] Y. Duan, S. Dou, S. Luo, H. Zhang, J. Lu, Adaptation of A-to-I RNA editing in Drosophila. PLOS Genet. 13, e1006648 (2017). 28282384 10.1371/journal.pgen.1006648 PMC5365144

[8] X. Li, I. M. Overton, R. A. Baines, L. P. Keegan, M. A. O’Connell, The ADAR RNA editing enzyme controls neuronal excitability in *Drosophila melanogaster*. Nucleic Acids Res. 42, 1139–1151 (2014). 24137011 10.1093/nar/gkt909 PMC3902911

[9] L. Grassi, G. Leoni, A. Tramontano, RNA editing differently affects protein-coding genes in D. melanogaster and H. sapiens. Sci. Rep. 5, 11550 (2015). 26169954 10.1038/srep11550 PMC4648400

[10] S. Garrett, J. J. C. Rosenthal, RNA editing underlies temperature adaptation in K^+^ channels from polar octopuses. Science 335, 848–851 (2012). 22223739 10.1126/science.1212795 PMC4219319

[11] M. Stapleton, J. W. Carlson, S. E. Celniker, RNA editing in *Drosophila melanogaster*: New targets and functional consequences. RNA 12, 1922–1932 (2006). 17018572 10.1261/rna.254306 PMC1624909

[12] M. Y. Ryan, R. Maloney, R. Reenan, R. Horn, Characterization of five RNA editing sites in Shab potassium channels. Channels 2, 202–209 (2008). 18836299 10.4161/chan.2.3.6386 PMC2783208

[13] M. Higuchi, S. Maas, F. N. Single, J. Hartner, A. Rozov, N. Burnashev, D. Feldmeyer, R. Sprengel, P. H. Seeburg, Point mutation in an AMPA receptor gene rescues lethality in mice deficient in the RNA-editing enzyme ADAR2. Nature 406, 78–81 (2000). 10894545 10.1038/35017558

[14] I. Gaisler-Salomon, E. Kravitz, Y. Feiler, M. Safran, A. Biegon, N. Amariglio, G. Rechavi, Hippocampus-specific deficiency in RNA editing of GluA2 in Alzheimer’s disease. Neurobiol. Aging 35, 1785–1791 (2014). 24679603 10.1016/j.neurobiolaging.2014.02.018

[15] M. T. Venø, J. B. Bramsen, C. Bendixen, F. Panitz, I. E. Holm, M. Öhman, J. Kjems, Spatio-temporal regulation of ADAR editing during development in porcine neural tissues. RNA Biol. 9, 1054–1065 (2012). 22858680 10.4161/rna.21082 PMC3551860

[16] H. Huang, B. Z. Tan, Y. Shen, J. Tao, F. Jiang, Y. Y. Sung, C. K. Ng, M. Raida, G. Köhr, M. Higuchi, H. Fatemi-Shariatpanahi, B. Harden, D. T. Yue, T. W. Soong, RNA editing of the IQ domain in Ca_v_1.3 channels modulates their Ca^2+^-dependent inactivation. Neuron 73, 304–316 (2012). 22284185 10.1016/j.neuron.2011.11.022 PMC3271027

[17] J. Zhai, S. Navakkode, S. Q. Z. Yeow, K. Krishna-K, M. C. Liang, J. H. Koh, R. X. Wong, W. P. Yu, S. Sajikumar, H. Huang, T. W. Soong, Loss of Ca1.3 RNA editing enhances mouse hippocampal plasticity, learning, and memory. Proc. Natl. Acad. Sci. U.S.A. 119, e2203883119 (2022). 35914168 10.1073/pnas.2203883119 PMC9371748

[18] L. Bazak, A. Haviv, M. Barak, J. Jacob-Hirsch, P. Deng, R. Zhang, F. J. Isaacs, G. Rechavi, J. B. Li, E. Eisenberg, E. Y. Levanon, A-to-I RNA editing occurs at over a hundred million genomic sites, located in a majority of human genes. Genome Res. 24, 365–376 (2014). 24347612 10.1101/gr.164749.113 PMC3941102

[19] Z. Peng, Y. Cheng, B. C.-M. Tan, L. Kang, Z. Tian, Y. Zhu, W. Zhang, Y. Liang, X. Hu, X. Tan, J. Guo, Z. Dong, Y. Liang, L. Bao, J. Wang, Comprehensive analysis of RNA-Seq data reveals extensive RNA editing in a human transcriptome. Nat. Biotechnol. 30, 253–260 (2012). 22327324 10.1038/nbt.2122

[20] G. Ramaswami, W. Lin, R. Piskol, M. H. Tan, C. Davis, J. B. Li, Accurate identification of human Alu and non-Alu RNA editing sites. Nat. Methods 9, 579–581 (2012). 22484847 10.1038/nmeth.1982 PMC3662811

[21] H. T. Porath, A. A. Schaffer, P. Kaniewska, S. Alon, E. Eisenberg, J. Rosenthal, E. Y. Levanon, O. Levy, A-to-I RNA editing in the earliest-diverging eumetazoan phyla. Mol. Biol. Evol. 34, 1890–1901 (2017). 28453786 10.1093/molbev/msx125 PMC5850803

[22] G. S. Laurent, M. R. Tackett, S. Nechkin, D. Shtokalo, D. Antonets, Y. A. Savva, R. Maloney, P. Kapranov, C. E. Lawrence, R. A. Reenan, Genome-wide analysis of A-to-I RNA editing by single-molecule sequencing in Drosophila. Nat. Struct. Mol. Biol. 20, 1333–1339 (2013). 24077224 10.1038/nsmb.2675

[23] B. R. Graveley, A. N. Brooks, J. W. Carlson, M. O. Duff, J. M. Landolin, L. Yang, C. G. Artieri, M. J. van Baren, N. Boley, B. W. Booth, J. B. Brown, L. Cherbas, C. A. Davis, A. Dobin, R. Li, W. Lin, J. H. Malone, N. R. Mattiuzzo, D. Miller, D. Sturgill, B. B. Tuch, C. Zaleski, D. Zhang, M. Blanchette, S. Dudoit, B. Eads, R. E. Green, A. Hammonds, L. Jiang, P. Kapranov, L. Langton, N. Perrimon, J. E. Sandler, K. H. Wan, A. Willingham, Y. Zhang, Y. Zou, J. Andrews, P. J. Bickel, S. E. Brenner, M. R. Brent, P. Cherbas, T. R. Gingeras, R. A. Hoskins, T. C. Kaufman, B. Oliver, S. E. Celniker, The developmental transcriptome of *Drosophila melanogaster*. Nature 471, 473–479 (2011). 21179090 10.1038/nature09715 PMC3075879

[24] K. Sinigaglia, D. Wiatrek, A. Khan, D. Michalik, N. Sambrani, J. Sedmík, D. Vukić, M. A. O’Connell, L. P. Keegan, ADAR RNA editing in innate immune response phasing, in circadian clocks and in sleep. Biochim. Biophys. Acta Gene Regul. Mech. 1862, 356–369 (2019). 30391332 10.1016/j.bbagrm.2018.10.011

[25] P. Deng, A. Khan, D. Jacobson, N. Sambrani, L. McGurk, X. Li, A. Jayasree, J. Hejatko, G. Shohat-Ophir, M. A. O’Connell, J. B. Li, L. P. Keegan, Adar RNA editing-dependent and -independent effects are required for brain and innate immune functions in *Drosophila*. Nat. Commun. 11, 1580 (2020). 32221286 10.1038/s41467-020-15435-1 PMC7101428

[26] M. J. Palladino, L. P. Keegan, M. A. O’Connell, R. A. Reenan, A-to-I pre-mRNA editing in *Drosophila* is primarily involved in adult nervous system function and integrity. Cell 102, 437–449 (2000). 10966106 10.1016/s0092-8674(00)00049-0

[27] Y. A. Savva, J. E. C. Jepson, A. Sahin, A. U. Sugden, J. S. Dorsky, L. Alpert, C. Lawrence, R. A. Reenan, Auto-regulatory RNA editing fine-tunes mRNA re-coding and complex behaviour in Drosophila. Nat. Commun. 3, 790 (2012). 22531175 10.1038/ncomms1789 PMC4017936

[28] J. E. C. Jepson, Y. A. Savva, C. Yokose, A. U. Sugden, A. Sahin, R. A. Reenan, Engineered alterations in RNA editing modulate complex behavior in *Drosophila:* Regulatory diversity of adenosine deaminase acting on RNA (ADAR) targets. J. Biol. Chem. 286, 8325–8337 (2011). 21078670 10.1074/jbc.M110.186817 PMC3048717

[29] J. E. Jepson, R. A. Reenan, Unraveling pleiotropic functions of A-to-I RNA editing in *Drosophila*. Fly 4, 154–158 (2010). 20215872 10.4161/fly.4.2.11232

[30] L. P. Keegan, J. Brindle, A. Gallo, A. Leroy, R. A. Reenan, M. A. O’Connell, Tuning of RNA editing by ADAR is required in *Drosophila*. EMBO J. 24, 2183–2193 (2005). 15920480 10.1038/sj.emboj.7600691 PMC1150885

[31] J. E. C. Jepson, R. A. Reenan, Adenosine-to-inosine genetic recoding is required in the adult stage nervous system for coordinated behavior in *Drosophila*. J. Biol. Chem. 284, 31391–31400 (2009). 19759011 10.1074/jbc.M109.035048 PMC2781535

[32] J. Ryvkin, A. Bentzur, A. Shmueli, M. Tannenbaum, O. Shallom, S. Dokarker, J. I. C. Benichou, M. Levi, G. Shohat-Ophir, Transcriptome analysis of NPFR neurons reveals a connection between Proteome diversity and social behavior. Front. Behav. Neurosci. 15, 628662 (2021). 33867948 10.3389/fnbeh.2021.628662 PMC8044454

[33] L. Ingleby, R. Maloney, J. Jepson, R. Horn, R. Reenan, Regulated RNA editing and functional epistasis in *Shaker* potassium channels. J. Gen. Physiol. 133, 17–27 (2009). 19114634 10.1085/jgp.200810133 PMC2606942

[34] A. K. Jones, S. D. Buckingham, M. Papadaki, M. Yokota, B. M. Sattelle, K. Matsuda, D. B. Sattelle, Splice-variant- and stage-specific RNA editing of the *Drosophila* GABA receptor modulates agonist potency. J. Neurosci. 29, 4287–4292 (2009). 19339622 10.1523/JNEUROSCI.5251-08.2009 PMC6665385

[35] R. O. Olson, Z. Liu, Y. Nomura, W. Song, K. Dong, Molecular and functional characterization of voltage-gated sodium channel variants from *Drosophila melanogaster*. Insect Biochem. Mol. Biol. 38, 604–610 (2008). 18405837 10.1016/j.ibmb.2008.01.003 PMC3056540

[36] M. Y. Ryan, R. Maloney, J. D. Fineberg, R. A. Reenan, R. Horn, RNA editing in eag potassium channels: Biophysical consequences of editing a conserved S6 residue. Channels 6, 443–452 (2012). 23064203 10.4161/chan.22314 PMC3536729

[37] A. L. Yablonovitch, J. Fu, K. Li, S. Mahato, L. Kang, E. Rashkovetsky, A. B. Korol, H. Tang, P. Michalak, A. C. Zelhof, E. Nevo, J. B. Li, Regulation of gene expression and RNA editing in Drosophila adapting to divergent microclimates. Nat. Commun. 8, 1570 (2017). 29146998 10.1038/s41467-017-01658-2 PMC5691062

[38] D. F. Cully, P. S. Paress, K. K. Liu, J. M. Schaeffer, J. P. Arena, Identification of a *Drosophila melanogaster* glutamate-gated chloride channel sensitive to the antiparasitic agent avermectin. J. Biol. Chem. 271, 20187–20191 (1996). 8702744 10.1074/jbc.271.33.20187

[39] A. Etter, D. F. Cully, J. M. Schaeffer, K. K. Liu, J. P. Arena, An amino acid substitution in the pore region of a glutamate-gated chloride channel enables the coupling of ligand binding to channel gating. J. Biol. Chem. 271, 16035–16039 (1996). 8663156 10.1074/jbc.271.27.16035

[40] W. W. Liu, R. I. Wilson, Glutamate is an inhibitory neurotransmitter in the *Drosophila* olfactory system. Proc. Natl. Acad. Sci. U.S.A. 110, 10294–10299 (2013). 23729809 10.1073/pnas.1220560110 PMC3690841

[41] A. J. Wolstenholme, Glutamate-gated chloride channels. J. Biol. Chem. 287, P40232–P40238 (2012). 10.1074/jbc.R112.406280 PMC3504739 23038250

[42] S. Kondo, T. Takahashi, N. Yamagata, Y. Imanishi, H. Katow, S. Hiramatsu, K. Lynn, A. Abe, A. Kumaraswamy, H. Tanimoto, Neurochemical organization of the *Drosophila* brain visualized by endogenously tagged neurotransmitter receptors. Cell Rep. 30, 284–297.e5 (2020). 31914394 10.1016/j.celrep.2019.12.018

[43] X. Li, C. Chien, Y. Han, Z. Sun, X. Chen, D. Dickman, Autocrine inhibition by a glutamate-gated chloride channel mediates presynaptic homeostatic depression. Sci. Adv. 7, eabj1215 (2021). 34851664 10.1126/sciadv.abj1215 PMC8635443

[44] L. N. Groschner, J. G. Malis, B. Zuidinga, A. Borst, A biophysical account of multiplication by a single neuron. Nature 603, 119–123 (2022). 35197635 10.1038/s41586-022-04428-3 PMC8891015

[45] Y. Li, P.-J. Chen, T.-Y. Lin, C.-Y. Ting, P. Muthuirulan, R. Pursley, M. Ilić, P. Pirih, M. S. Drews, K. P. Menon, K. G. Zinn, T. Pohida, A. Borst, C.-H. Lee, Neural mechanism of spatio-chromatic opponency in the *Drosophila* amacrine neurons. Curr. Biol. 31, 3040–3052.e9 (2021). 34033749 10.1016/j.cub.2021.04.068

[46] R. Zhang, P. Deng, D. Jacobson, J. B. Li, Evolutionary analysis reveals regulatory and functional landscape of coding and non-coding RNA editing. PLOS Genet. 13, e1006563 (2017). 28166241 10.1371/journal.pgen.1006563 PMC5319793

[47] A. L. Sapiro, A. Shmueli, G. L. Henry, Q. Li, T. Shalit, O. Yaron, Y. Paas, J. Billy Li, G. Shohat-Ophir, Illuminating spatial A-to-I RNA editing signatures within the *Drosophila* brain. Proc. Natl. Acad. Sci. U.S.A. 116, 2318–2327 (2019). 30659150 10.1073/pnas.1811768116 PMC6369821

[48] F. Teufel, J. J. Almagro Armenteros, A. R. Johansen, M. H. Gíslason, S. I. Pihl, K. D. Tsirigos, O. Winther, S. Brunak, G. von Heijne, H. Nielsen, SignalP 6.0 predicts all five types of signal peptides using protein language models. Nat. Biotechnol. 40, 1023–1025 (2022). 34980915 10.1038/s41587-021-01156-3 PMC9287161

[49] D. T. Jones, Protein secondary structure prediction based on position-specific scoring matrices. J. Mol. Biol. 292, 195–202 (1999). 10493868 10.1006/jmbi.1999.3091

[50] T. Nugent, D. T. Jones, Transmembrane protein topology prediction using support vector machines. BMC Bioinformatics 10, 159 (2009). 19470175 10.1186/1471-2105-10-159 PMC2700806

[51] D. W. A. Buchan, D. T. Jones, The PSIPRED protein analysis workbench: 20 years on. Nucleic Acids Res. 47, W402–W407 (2019). 31251384 10.1093/nar/gkz297 PMC6602445

[52] A. Drozdetskiy, C. Cole, J. Procter, G. J. Barton, JPred4: A protein secondary structure prediction server. Nucleic Acids Res. 43, W389–WW94 (2015). 25883141 10.1093/nar/gkv332 PMC4489285

[53] F. Sievers, D. G. Higgins, Clustal Omega for making accurate alignments of many protein sequences. Protein Sci. 27, 135–145 (2018). 28884485 10.1002/pro.3290 PMC5734385

[54] S. Rajkumar, T. M. Böckers, A. Catanese, Fast and efficient synaptosome isolation and post-synaptic density enrichment from hiPSC-motor neurons by biochemical sub-cellular fractionation. STAR Protoc 4, 102061 (2023). 36853677 10.1016/j.xpro.2023.102061 PMC9898788

[55] L. Buck, R. Axel, A novel multigene family may encode odorant receptors: A molecular basis for odor recognition. Cell 65, 175–187 (1991). 1840504 10.1016/0092-8674(91)90418-x

[56] L. B. Vosshall, H. Amrein, P. S. Morozov, A. Rzhetsky, R. Axel, A spatial map of olfactory receptor expression in the Drosophila antenna. Cell 96, 725–736 (1999). 10089887 10.1016/s0092-8674(00)80582-6

[57] E. A. Hallem, M. G. Ho, J. R. Carlson, The molecular basis of odor coding in the Drosophila antenna. Cell 117, 965–979 (2004). 15210116 10.1016/j.cell.2004.05.012

[58] D. Task, C.-C. Lin, A. Vulpe, A. Afify, S. Ballou, M. Brbic, P. Schlegel, J. Raji, G. Jefferis, H. Li, K. Menuz, C. J. Potter, Chemoreceptor co-expression in *Drosophila melanogaster* olfactory neurons. eLife 11, e72599 (2022). 35442190 10.7554/eLife.72599 PMC9020824

[59] Q. Gao, B. Yuan, A. Chess, Convergent projections of *Drosophila* olfactory neurons to specific glomeruli in the antennal lobe. Nat. Neurosci. 3, 780–785 (2000). 10903570 10.1038/77680

[60] P. Mombaerts, F. Wang, C. Dulac, S. K. Chao, A. Nemes, M. Mendelsohn, J. Edmondson, R. Axel, Visualizing an olfactory sensory map. Cell 87, 675–686 (1996). 8929536 10.1016/s0092-8674(00)81387-2

[61] L. B. Vosshall, A. M. Wong, R. Axel, An olfactory sensory map in the fly brain. Cell 102, 147–159 (2000). 10943836 10.1016/s0092-8674(00)00021-0

[62] N. K. Tanaka, K. Endo, K. Ito, Organization of antennal lobe-associated neurons in adult *Drosophila melanogaster* brain. J. Comp. Neurol. 520, 4067–4130 (2012). 22592945 10.1002/cne.23142

[63] A. Schneider, M. Ruppert, O. Hendrich, T. Giang, M. Ogueta, S. Hampel, M. Vollbach, A. Büschges, H. Scholz, Neuronal basis of innate olfactory attraction to ethanol in *Drosophila*. PLOS ONE 7, e52007 (2012). 23284851 10.1371/journal.pone.0052007 PMC3527413

[64] A. V. Devineni, U. Heberlein, Preferential ethanol consumption in *Drosophila* models features of addiction. Curr. Biol. 19, 2126–2132 (2009). 20005106 10.1016/j.cub.2009.10.070 PMC2805771

[65] S.-P. Wang, W.-Y. Guo, S. A. Muhammad, R.-R. Chen, L.-L. Mu, G.-Q. Li, Mating experience and food deprivation modulate odor preference and dispersal in *Drosophila melanogaster* males. J. Insect Sci. 14, 131 (2014). 25368075 10.1093/jis/14.1.131 PMC4222301

[66] R. Azanchi, K. R. Kaun, U. Heberlein, Competing dopamine neurons drive oviposition choice for ethanol in *Drosophila*. Proc. Natl. Acad. Sci. U.S.A. 110, 21153–21158 (2013). 24324162 10.1073/pnas.1320208110 PMC3876210

[67] M. Parnas, A. C. Lin, W. Huetteroth, G. Miesenböck, Odor discrimination in *Drosophila*: From neural population codes to behavior. Neuron 79, 932–944 (2013). 24012006 10.1016/j.neuron.2013.08.006 PMC3765961

[68] M. dela Paz Fernández, Y.-B. Chan, J. Y. Yew, J.-C. Billeter, K. Dreisewerd, J. D. Levine, E. A. Kravitz, Pheromonal and behavioral cues trigger male-to-female aggression in *Drosophila*. PLoS Biol. 8, e1000541 (2010). 21124886 10.1371/journal.pbio.1000541 PMC2990703

[69] A. Bentzur, A. Shmueli, L. Omesi, J. Ryvkin, J.-M. Knapp, M. Parnas, F. P. Davis, G. Shohat-Ophir, Odorant binding protein *69a* connects social interaction to modulation of social responsiveness in *Drosophila*. PLOS Genet. 14, e1007328 (2018). 29630598 10.1371/journal.pgen.1007328 PMC5908198

[70] D. P. Smith, Odor and pheromone detection in *Drosophila melanogaster*. Pflugers Arch. 454, 749–758 (2007). 17205355 10.1007/s00424-006-0190-2

[71] L. Wang, D. J. Anderson, Identification of an aggression-promoting pheromone and its receptor neurons in *Drosophila*. Nature 463, 227–231 (2010). 19966787 10.1038/nature08678 PMC2999963

[72] R. M. Joseph, A. V. Devineni, I. F. G. King, U. Heberlein, Oviposition preference for and positional avoidance of acetic acid provide a model for competing behavioral drives in *Drosophila*. Proc. Natl. Acad. Sci. U.S.A. 106, 11352–11357 (2009). 19541615 10.1073/pnas.0901419106 PMC2698888

[73] J. A. Riffell, Neuroethology: Lemon-fresh scent makes flies lay eggs. Curr. Biol. 23, R1108–R1110 (2013). 24355790 10.1016/j.cub.2013.11.003

[74] S.-L. Lai, T. Awasaki, K. Ito, T. Lee, Clonal analysis of *Drosophila* antennal lobe neurons: Diverse neuronal architectures in the lateral neuroblast lineage. Development 135, 2883–2893 (2008). 18653555 10.1242/dev.024380

[75] J. J. C. Rosenthal, The emerging role of RNA editing in plasticity. J. Exp. Biol. 218, 1812–1821 (2015). 26085659 10.1242/jeb.119065 PMC4487009

[76] G. Galarza-Muñoz, S. I. Soto-Morales, M. Holmgren, J. J. C. Rosenthal, Physiological adaptation of an Antarctic Na^+^/K^+^-ATPase to the cold. J. Exp. Biol. 214, 2164–2174 (2011). 21653810 10.1242/jeb.048744 PMC3110501

[77] J. J. C. Rosenthal, P. H. Seeburg, A-to-I RNA editing: Effects on proteins key to neural excitability. Neuron 74, 432–439 (2012). 22578495 10.1016/j.neuron.2012.04.010 PMC3724421

[78] S. Alon, S. C. Garrett, E. Y. Levanon, S. Olson, B. R. Graveley, J. J. C. Rosenthal, E. Eisenberg, The majority of transcripts in the squid nervous system are extensively recoded by A-to-I RNA editing. eLife 4, e05198 (2015). 25569156 10.7554/eLife.05198 PMC4384741

[79] J. Alcedo, C. Kenyon, Regulation of C. elegans longevity by specific gustatory and olfactory neurons. Neuron 41, 45–55 (2004). 14715134 10.1016/s0896-6273(03)00816-x

[80] J. Apfeld, C. Kenyon, Regulation of lifespan by sensory perception in *Caenorhabditis elegans*. Nature 402, 804–809 (1999). 10617200 10.1038/45544

[81] L. Badel, K. Ohta, Y. Tsuchimoto, H. Kazama, Decoding of context-dependent olfactory behavior in *Drosophila*. Neuron 91, 155–167 (2016). 27321924 10.1016/j.neuron.2016.05.022

[82] Y. Kondoh, K. Y. Kaneshiro, K.-I. Kimura, D. Yamamoto, Evolution of sexual dimorphism in the olfactory brain of Hawaiian *Drosophila*. Proc. Biol. Sci. 270, 1005–1013 (2003). 12803889 10.1098/rspb.2003.2331 PMC1691346

[83] P. Stockinger, D. Kvitsiani, S. Rotkopf, L. Tirián, B. J. Dickson, Neural circuitry that governs *Drosophila* male courtship behavior. Cell 121, 795–807 (2005). 15935765 10.1016/j.cell.2005.04.026

[84] A. Sakurai, M. Koganezawa, K.-I. Yasunaga, K. Emoto, D. Yamamoto, Select interneuron clusters determine female sexual receptivity in *Drosophila*. Nat. Commun. 4, 1825 (2013). 23652013 10.1038/ncomms2837 PMC3674241

[85] M. L. Schlief, R. I. Wilson, Olfactory processing and behavior downstream from highly selective receptor neurons. Nat. Neurosci. 10, 623–630 (2007). 17417635 10.1038/nn1881 PMC2838507

[86] S. R. Olsen, V. Bhandawat, R. I. Wilson, Excitatory interactions between olfactory processing channels in the *Drosophila* antennal lobe. Neuron 54, 89–103 (2007). 17408580 10.1016/j.neuron.2007.03.010 PMC2048819

[87] R. Brusa, F. Zimmermann, D. S. Koh, D. Feldmeyer, P. Gass, P. H. Seeburg, R. Sprengel, Early-onset epilepsy and postnatal lethality associated with an editing-deficient *GluR*-*B* allele in mice. Science 270, 1677–1680 (1995). 7502080 10.1126/science.270.5242.1677

[88] C. Gonzalez, A. Lopez-Rodriguez, D. Srikumar, J. J. C. Rosenthal, M. Holmgren, Editing of human K_V_1.1 channel mRNAs disrupts binding of the N-terminus tip at the intracellular cavity. Nat. Commun. 2, 436 (2011). 21847110 10.1038/ncomms1446 PMC3265383

[89] R. E. Hibbs, E. Gouaux, Principles of activation and permeation in an anion-selective Cys-loop receptor. Nature 474, 54–60 (2011). 21572436 10.1038/nature10139 PMC3160419

[90] J. Yu, H. Zhu, R. Lape, T. Greiner, J. Du, W. Lü, L. Sivilotti, E. Gouaux, Mechanism of gating and partial agonist action in the glycine receptor. Cell 184, 957–968.e21 (2021). 33567265 10.1016/j.cell.2021.01.026 PMC8115384

[91] G. Ramaswami, P. Deng, R. Zhang, M. Anna Carbone, T. F. C. Mackay, J. Billy Li, Genetic mapping uncovers cis-regulatory landscape of RNA editing. Nat. Commun. 6, 8194 (2015). 26373807 10.1038/ncomms9194 PMC4573499

[92] J. Cox, M. Y. Hein, C. A. Luber, I. Paron, N. Nagaraj, M. Mann, Accurate proteome-wide label-free quantification by delayed normalization and maximal peptide ratio extraction, termed MaxLFQ. Mol. Cell. Proteomics 13, 2513–2526 (2014). 24942700 10.1074/mcp.M113.031591 PMC4159666

[93] M. Kabra, A. A. Robie, M. Rivera-Alba, S. Branson, K. Branson, JAABA: Interactive machine learning for automatic annotation of animal behavior. Nat. Methods 10, 64–67 (2013). 23202433 10.1038/nmeth.2281

[94] A. Bentzur, S. Ben-Shaanan, J. I. C. Benichou, E. Costi, M. Levi, A. Ilany, G. Shohat-Ophir, Early life experience shapes male behavior and social networks in *Drosophila*. Curr. Biol. 31, 486–501.e3 (2021). 33186552 10.1016/j.cub.2020.10.060

[95] J. E. Manoim, A. M. Davidson, S. Weiss, T. Hige, M. Parnas, Lateral axonal modulation is required for stimulus-specific olfactory conditioning in *Drosophila*. Curr. Biol. 32, 4438–4450.e5 (2022). 36130601 10.1016/j.cub.2022.09.007 PMC9613607

[96] E. Rozenfeld, H. Lerner, M. Parnas, Muscarinic modulation of antennal lobe GABAergic local neurons shapes odor coding and behavior. Cell Rep. 29, 3253–3265.e4 (2019). 31801087 10.1016/j.celrep.2019.10.125 PMC6900217

[97] S. Israel, E. Rozenfeld, D. Weber, W. Huetteroth, M. Parnas, Olfactory stimuli and moonwalker SEZ neurons can drive backward locomotion in *Drosophila*. Curr. Biol. 32, 1131–1149.e7 (2022). 35139358 10.1016/j.cub.2022.01.035 PMC8926844

[98] E. Rozenfeld, M. Tauber, Y. Ben-Chaim, M. Parnas, GPCR voltage dependence controls neuronal plasticity and behavior. Nat. Commun. 12, 7252 (2021). 34903750 10.1038/s41467-021-27593-x PMC8668892

[99] E. Rozenfeld, N. Ehmann, J. E. Manoim, R. J. Kittel, M. Parnas, Homeostatic synaptic plasticity rescues neural coding reliability. Nat. Commun. 14, 2993 (2023). 37225688 10.1038/s41467-023-38575-6 PMC10209050

