# Supplementary material for: A highly conserved A-to-I RNA editing event within the glutamate-gated chloride channel GluClα is necessary for olfactory-based behaviors in Drosophila
Source: Sci Adv. 2024 Sep 4;10(36):eadi9101. doi: 10.1126/sciadv.adi9101 (PMC11373593; doi:10.1126/sciadv.adi9101)
Supplement: Supplementary file 1 — Figs. S1 to S5 Legends for tables S1 to S6 Table S7 [file sciadv.adi9101_sm.pdf]

Science Advances
AAAS

Supplementary Materials for

A highly conserved A-to-I RNA editing event within the glutamate-gated chloride channel GluClα is necessary for olfactory-based behaviors in *Drosophila*

Hila Zak *et al.*

Corresponding author: Galit Shohat-Ophir, galit.ophir@biu.ac.il

Sci. Adv. **10**, eadi9101 (2024)
DOI: 10.1126/sciadv.adi9101

The PDF file includes:

Figs. S1 to S5
Legends for tables S1 to S6
Table S7

Other Supplementary Material for this manuscript includes the following:

Tables S1 to S6

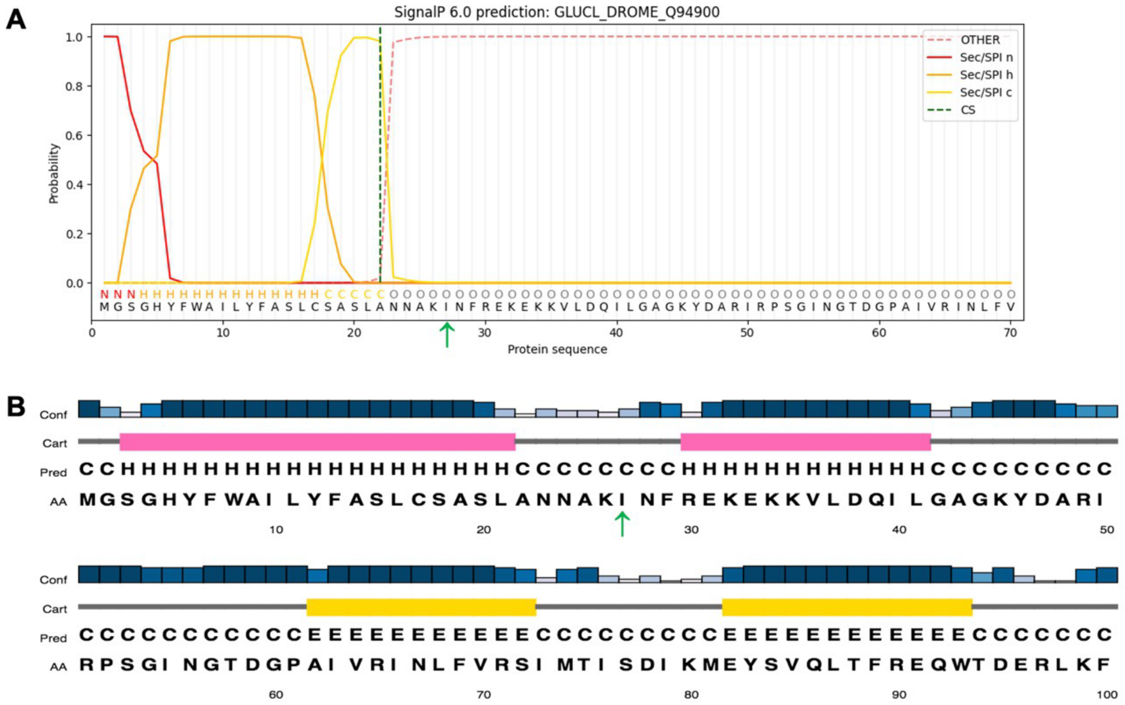

C

```
GLUCL_DROME (Q94900)  -----------------MGSGHYFWAILYF------------------------------   13
GLUCL_CAEEL (G5EBR3)  ---------------------MATWIVGKLIIASLILGIQAQQARTKSQDIFEDDNDNGT   39
GBRA1_RAT (P62813)    -----------MKKSRGLSDYLWAWTLILSTL-------SGRSYGQPSQDELKDNT----   38
GBRB1_RAT (P15431)    -----------------------MWTVQNRE--------SLGLLSFPVMVAMVCCAHSSN   29
GBRG2_RAT (P18508)    MSSPNTWSTGSTVYSPVFSQKMTLWILLLLSL-------YPGFTSQKSDDDYEDYASNKT   53
GLRA3_HUMAN (O75311)  --------MAHVRHFRTLVSGFYFWEAALLLS-------------------LVATKET--   31
GLRA1_DANRE (O93430)  ----------------MFALGIYLWETIVFFS-------------------LAASQQA--   23
GLRB2_DANRE (Q6DC22)  ---------MKALKVIFMLLIICLWMEGGFTKEK-----SAKKGKKKGKQVYCPSQLS--   44
GLRA1_PIG (F1RQB7)    -------------MYRFNTLRLYLWETIVFFS-------------------LAASKEA--   26
GLRA2_HUMAN (P23416)  --------MNRQLVNILTALFAFFLETNHFRT------------------AFCKDHDS--   32
GLRB_HUMAN (P48167)   ---------MKFLLTTAFLILISLWVEEAYSKEK-----SSKKGKGKKKQYLCPSQQS--   44

GLUCL_DROME (Q94900)  ----ASLCSASLANNAKINFREKEKKVLDQILGA-GKYDARIRPSGINGTDGPAIVRINL   68
GLUCL_CAEEL (G5EBR3)  TTLESLARLTSPIHIPIEQPQTSDSKILAHLFT--SGYDFRVRPPT--DNGGPVVVSVNM   95 [3RIF]
GBRA1_RAT (P62813)    ---------------------TVFTRILDRLL---DGYDNRLRPGL---GERVTEVKTDI   71 [6DW0]
GBRB1_RAT (P15431)    ----------------EPSNMSYVKETVDRLL---KGYDIRLRPDF---GGPPVDVGMRI   67 [6DW0]
GBRG2_RAT (P18508)    ------------WVLTPKVPEGDVTVILNNLL---EGYDNKLRPDI---GVKPTLIHTDM   95 [6DW0]
GLRA3_HUMAN (O75311)  ------------DSARSRSAPMSPSDFLDKLMGRTSGYDARIRPNF---KGPPVNVTCNI   76 [5CFB]
GLRA1_DANRE (O93430)  -------------AARKAASPMPPSEFLDKLMGKVSGYDARIRPNF---KGPPVNVTCNI   67 [6PLR]
GLRB2_DANRE (Q6DC22)  ------------SEDLARVPANSTSNILNKLL---ITYDPRIRPNF---KGIPVEDRVNI   86 [7TVI]
GLRA1_PIG (F1RQB7)    ------------EAARSASKPMSPSDFLDKLMGRTSGYDARIRPNF---KGPPVNVSCNI   71 [7MLU]
GLRA2_HUMAN (P23416)  ------------RSGKQPSQTLSPSDFLDKLMGRTSGYDARIRPNF---KGPPVNVTCNI   77 [7KUY]
GLRB_HUMAN (P48167)   ------------AEDLARVPANSTSNILNRLL---VSYDPRIRPNF---KGIPVDVVVNI   86 [5BKF]
                                              .  : .::     ** ::**         .     :
```

Supplementary Figure 1. Location of the I27V editing site in the *Drosophila* GluClα subunit (UniProt ID Q94900). (**A**) SignalP 6.0 prediction showing that the probability of the presence of a signal-peptide cleavage site (CS) between positions 22 and 23 is $P = 0.98$. The Secretory (Sec) signal peptide n-region (Sec/SPI n) corresponds to the n-terminal region of the signal peptide and is labeled as N (above the amino acid sequence). Sec/SPI h corresponds to the center hydrophobic region of the signal peptide and is labeled as H. Sec/SPI c corresponds to the c-terminal region of the signal peptide and is labeled as C. OTHER corresponds to a sequence other than a signal peptide and is labeled as O. I27 is indicated by a green arrow. **(B)** PSIPRED 4.0 secondary structure

prediction for the *Drosophila* GluClα immature protein. The full-length protein sequence was submitted for the prediction, whereas only the first 100 amino acids are shown. The protein secondary structure prediction is based on position-specific scoring matrices (PSSMs). Helices (H), strands (E) and coils (C) are indicated in pink, yellow and gray colors, respectively. Conf, confidence of prediction with the highest confidence in dark blue. AA, the target sequence. I27 is indicated by a green arrow. Note that transmembrane (TM) protein topology prediction based on support vector machines (SVM) (MEMSAT-SVM in the PSIPRED server) predicted amino acids 2-20 of the immature protein to be a signal peptide α-helix (not shown). **(C)** Iterative multiple sequence alignment between the *Drosophila* GluClα subunit (UniProt ID Q94900) and various subunits of anionic Cys-loop receptors whose three-dimensional (3-D) structure was determined at high resolution by X-ray crystallography or cryo-electron microscopy. For clarity, merely an N-terminal portion of the sequences is presented. The UniProt names and identifiers are indicated on the left side of the sequences. The PDB ID codes that correspond to the 3-D structures of the recombinant Cys-loop anionic receptors are indicated in square parentheses on the right side of the sequences. Signal peptides are underlined in red according to the UniProt Knowledgebase (UniProtKB). The edited amino acid (I27) is boxed in green and indicated by a green arrow. The first amino acid of each 3-D structure is boxed in cyan. Helix α1 is shown in white letters on pink background. The highly conserved amino-acid cluster mentioned in the manuscript text is indicated by a gray background. The beginning of the first beta strand (Ω1) is indicated by a yellow background. Asterisk indicates positions which have a single, fully conserved residue. Colon indicates conservation between groups of strongly similar properties. Period indicates conservation between groups of weakly similar properties.

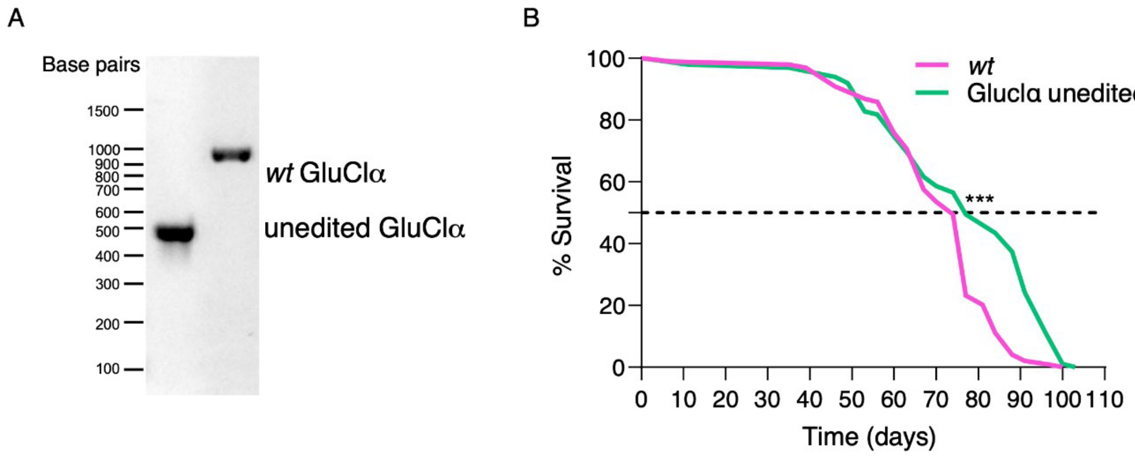

Supplementary Figure 2. GluClα unedited flies have extended lifespan. (A) PCR validation of ECS deletion in GluClα unedited and *wt* flies. The size of the segment in wild-type flies is ~900bp whereas in GluClα unedited flies it is ~490bp. (A) 99 flies of *wt* (purple) and GluClα unedited flies (green) were housed singly and the number of live flies in both cohorts was compared twice a week. ***P<0.001, Log-rank (Mantel-Cox) test.

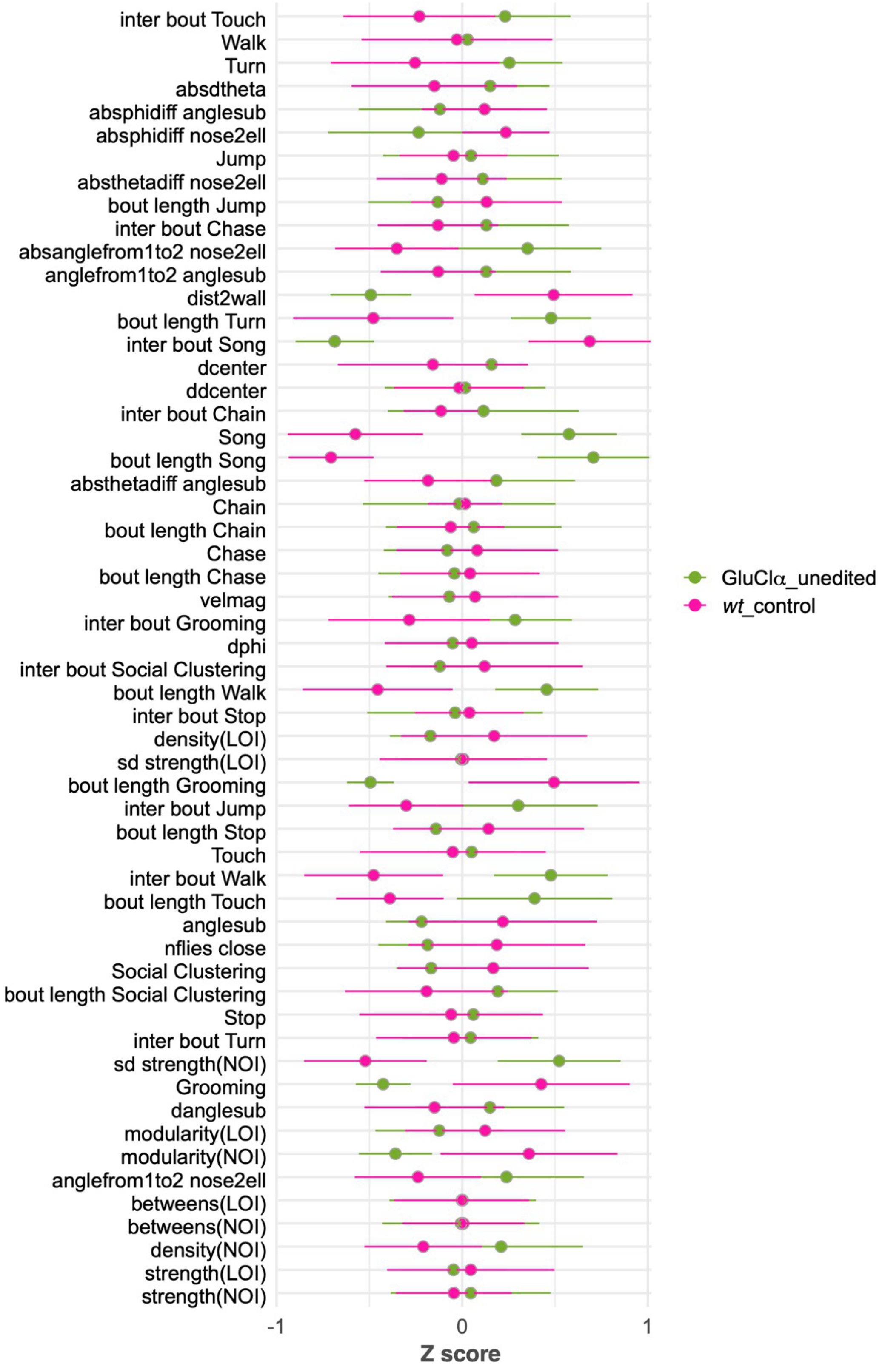

Supplementary Figure 3. GluClα unedited flies show normal kinetic and social parameters in the FlyBowl system. Data are represented as normalized Z scores of 60 behavioral parameters, n = 7. No significant differences between GluClα unedited and *wt* male flies. Statistical analysis was determined by one-way ANOVA followed by Tukey's range test for experiments that were distributed normally, and by Kruskal–Wallis test followed by Wilcoxon signed-rank test for experiments that were not distributed normally. FDR correction was used for multiple tests.

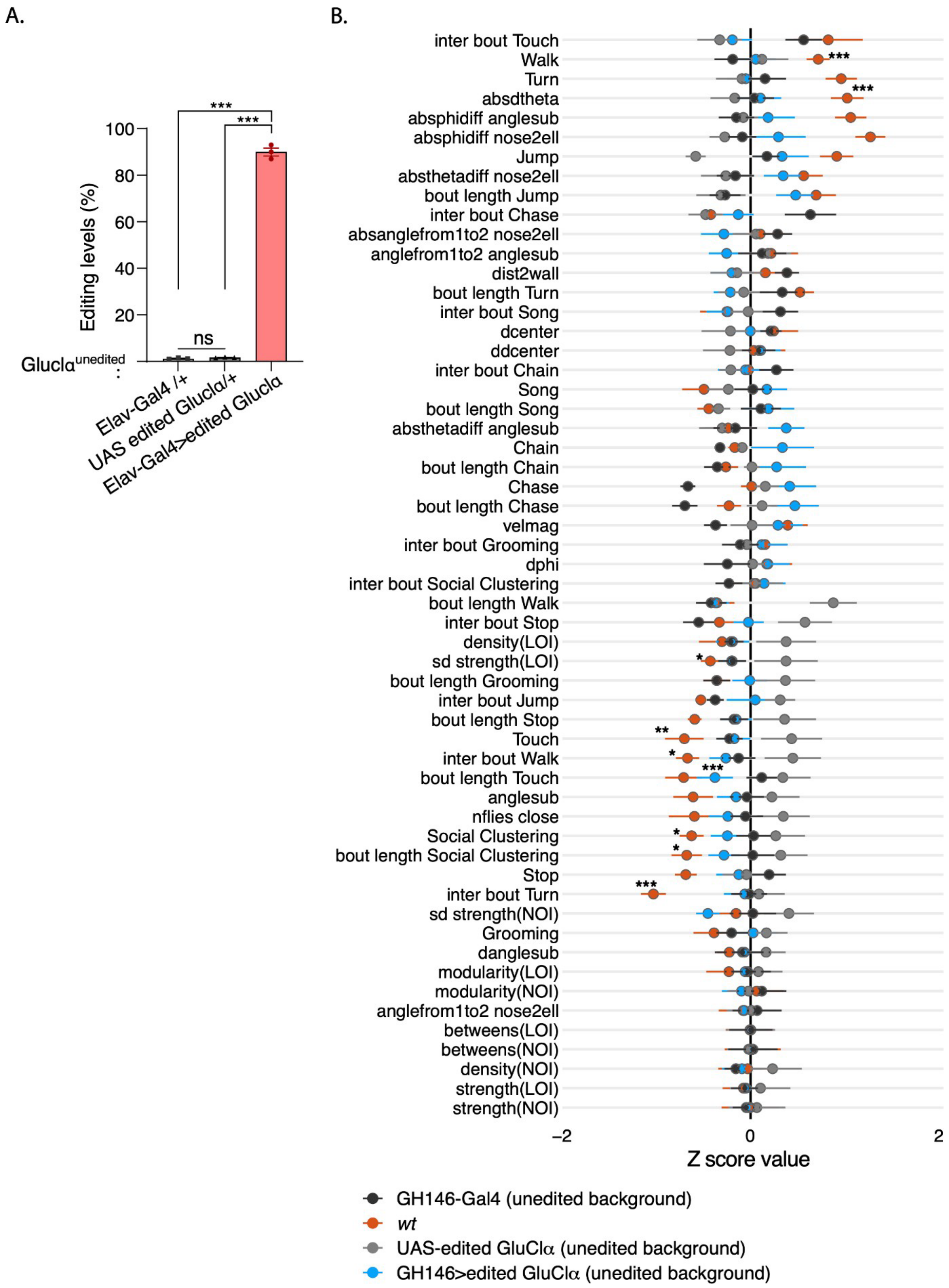

Supplementary Figure 4. Expression of GluClα edited channel restores editing levels and does not affect motor responses. (A) Relative editing levels of site 15579486 I27V was determined in GluClα unedited files expressing the edited GluClα using a pan-neuronal driver and the genetic controls using Sanger sequencing. n=3, ***P<0.001 one-way ANOVA. (B) GluClα unedited flies expressing fully edited channel in PNs exhibit normal motor and social responses compared to genetic controls and *wt* flies. Data are represented as normalized Z scores of 60 behavioral parameters, n = 8. Statistical analysis was determined by one-way ANOVA followed by Tukey's range test for experiments that were distributed normally, and by Kruskal–Wallis test followed by Wilcoxon signed-rank test for experiments that were not distributed normally. FDR correction was used for multiple tests.

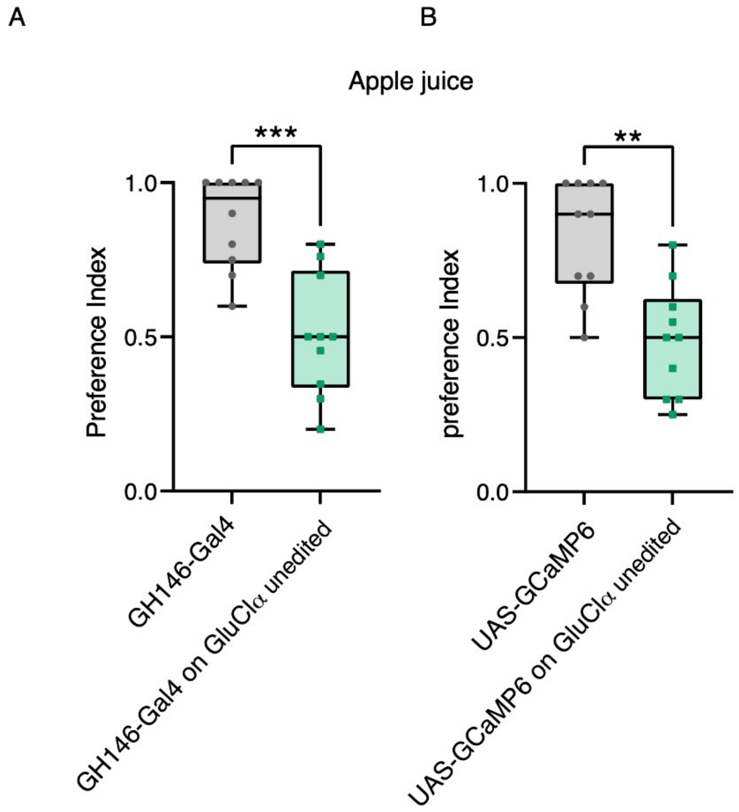

Supplementary Figure 5. Validation strains for calcium imaging. (A) Preference of GH146-Gal4 lines and GH146-Gal4 on GluClα unedited males towards apple juice. n=10 ***P<0.0001; Mann-Whitney test. (B) Preference of UAS-GCaMP6 lines and UAS-GCaMP6 on GluClα unedited males towards apple juice. n=10 ***P<0.001; Mann-Whitney test.

Definition	Description
Walk	Fly moves.
Stop	Fly is still.
Turn	Changes in fly's direction.
Touch	Fly actively touches another fly.
Social cluster	Fly sits in a group of 3 or more flies.
Long distance Approach	Fly approaches from a distance another fly and perform interaction.
Short distance approach	Fly performs an interaction during when in social cluster.
Grooming	Fly grooms.
Chase	Fly chases another fly.
Chain	Chase with 3 or more flies.
Song	Fly moves one wing next to another fly.
Behavior bout length	Length of the longest sequence of frames in which the behavior occurred per fly.
Behavior inter bout length	Duration between bouts of same behavior.
dnose2ell	Minimum distance from any point of this animal nose to the ellipse of other flies.
absanglefrom1to2nose2ell	Absolute difference between direction to closest animal and current animal's orientation (rad).
absdtheta	Angular speed (rad/s).
absphidiff anglesub	Absolute difference in velocity direction between current animal and closest animal(rad).
absphidiff nose2ell	Absolute difference in velocity direction between current animal and closest animal (rad).
absthetadiff anglesub	Absolute difference in orientation between current animal and closest animal (rad).
absthetadiff nose2ell	Absolute difference in orientation between this animal and closest animal (rad).
anglefrom1to2 anglesub	Angle to closest animal's centroid in current animal's coordinate system (rad).
anglefrom1to2 nose2ell	Angle to closest animal's centroid in current animal's coordinate system (rad).
angleonclosestfly	Angle of the current animal's centroid in the closest animal's coordinate system (rad).
anglesub	Maximum total angle of animal's field of view (fov) occluded by another animal (rad).
danglesub	Change in maximum total angle of animal's view occluded by another animal (rad/s).
dcenter	Minimum distance from this animal's center to other animal's center (mm).
ddcenter	Change in minimum distance between this animal's center and other flies' centers (mm/s).
dist2wall	Distance to the arena wall from the animal's center (mm).
dphi	Change in the velocity direction (rad/s).
dtheta	Angular velocity (rad/s).
nflies_close	Number of flies within 2 body lengths (4a).
velmag	Speed of the center of rotation (mm/s).
Density SD by length of interactions (LOI)	Accumulated interactions' length relative to the maximum interactions' length possible.
Modularity by length of interactions (LOI)	Representation of how much the network is divided into modules according to interactions' length.
Strength by length of interactions (LOI)	Length of interactions of a certain fly.
SD Strength according to length of interactions (LOI)	Standard divination of the strengths according to interactions' length of flies from the same movie.
Betweenness Centrality by length of interactions (LOI)	A measure of centrality of a certain fly based on shortest paths according to interactions' length.
SD Betweenness Centrality by length of interactions (LOI)	Standard divination of the betweenness centralities according to interactions' length of flies from the same movie.
Density by number of interactions (NOI)	Interactions' number relative to the maximum interactions' number possible.
Modularity Strength by number of interactions (NOI)	Representation of how much the network is divided into modules according to interactions' number.
Strength by number of interactions (NOI)	Number of interactions of a certain fly.
SD Strength by number of interactions (NOI)	Standard divination of the strengths according to interactions' number of flies from the same movie.
Betweenness Centrality by number of interactions (NOI)	A measure of centrality of a certain fly based on shortest paths according to interactions' number.
SD Betweenness centrality (by number of interactions (NOI)	Variance of the betweenness centralities according to interactions' number of flies from the same movie.

Supplementary Table 7. A list of the features and behaviors measured by the FlyBowl system. Explanation to the features depicted in S3, 5

Supp table 1: Raw data of Fig. 1B

Supp table 2a: Raw data of Fig. 1C

Supp table 2b: Raw data of Fig. 1C

Supp table 3: Raw data of Fig. 1E

Supp table 4: Raw data of Fig. 1G

Supp table 5: Raw data of Fig. S3

Supp table 6: Raw data of Fig. S4B

Supp table 7: List of the features and behaviors measured by the FlyBowl system depicted in Fig. S3, S4B.
